# Comparison of Small Gut and Whole Gut Microbiota of First-Degree Relatives With Adult Celiac Disease Patients and Controls

**DOI:** 10.3389/fmicb.2019.00164

**Published:** 2019-02-08

**Authors:** Rahul Bodkhe, Sudarshan A. Shetty, Dhiraj P. Dhotre, Anil K. Verma, Khushbo Bhatia, Asha Mishra, Gurvinder Kaur, Pranav Pande, Dhinoth K. Bangarusamy, Beena P. Santosh, Rajadurai C. Perumal, Vineet Ahuja, Yogesh S. Shouche, Govind K. Makharia

**Affiliations:** ^1^National Centre for Microbial Resource, National Centre for Cell Science, Pune, India; ^2^Department of Gastroenterology and Human Nutrition, All India Institute of Medical Sciences, New Delhi, India; ^3^Department of Transplant Immunology and Immunogenetics, All India Institute of Medical Sciences, New Delhi, India; ^4^AgriGenome Labs Pvt. Ltd., Kerala, India

**Keywords:** celiac, gut microbiota, gluten, *H. pylori*, butyrate, duodenal microbiota

## Abstract

Recent studies on celiac disease (CeD) have reported alterations in the gut microbiome. Whether this alteration in the microbial community is the cause or effect of the disease is not well understood, especially in adult onset of disease. The first-degree relatives (FDRs) of CeD patients may provide an opportunity to study gut microbiome in pre-disease state as FDRs are genetically susceptible to CeD. By using 16S rRNA gene sequencing, we observed that ecosystem level diversity measures were not significantly different between the disease condition (CeD), pre-disease (FDR) and control subjects. However, differences were observed at the level of amplicon sequence variant (ASV), suggesting alterations in specific ASVs between pre-disease and diseased condition. Duodenal biopsies showed higher differences in ASVs compared to fecal samples indicating larger disruption of the microbiota at the disease site. The duodenal microbiota of FDR was characterized by significant abundance of ASVs belonging to *Parvimonas, Granulicatella, Gemella, Bifidobacterium, Anaerostipes*, and *Actinomyces* genera. The duodenal microbiota of CeD was characterized by higher abundance of ASVs from genera *Megasphaera* and *Helicobacter* compared to the FDR microbiota. The CeD and FDR fecal microbiota had reduced abundance of ASVs classified as *Akkermansia* and *Dorea* when compared to control group microbiota. In addition, predicted functional metagenome showed reduced ability of gluten degradation by CeD fecal microbiota in comparison to FDRs and controls. The findings of the present study demonstrate differences in ASVs and predicts reduced ability of CeD fecal microbiota to degrade gluten compared to the FDR fecal microbiota. Further research is required to investigate the strain level and active functional profiles of FDR and CeD microbiota to better understand the role of gut microbiome in pathophysiology of CeD.

## Introduction

Celiac disease is a common, chronic immune mediated enteropathy of the small intestine which affects approximately 0.7% of the global population (Singh et al., 2018). Once thought to be uncommon in Asia, CeD is now prevalent in many Asian countries including India (Makharia et al., 2011). Recently, the prevalence of CeD has been on the rise, especially in developing countries (Lohi et al., 2007). This rapid rise in disease prevalence cannot be attributed only to the underlying genetic makeup of the population but rather to the environmental factors including infant feeding practices, reduction in infectious diseases, reovirus infection, and use of antibiotics (Jabri and Sollid, 2006; Lohi et al., 2007; Myléus et al., 2009; Volta and De Giorgio, 2012; Mårild et al., 2013; Bouziat et al., 2017).

CeD is caused by the consumption of gluten proteins present in cereals such as wheat, barley and rye in genetically susceptible individuals (Caminero et al., 2015). While many genes are involved in the development of CeD, thus far only the presence of HLA-DQ2 or DQ8 haplotype is considered to be essential (Sanz et al., 2011). Additional factors that contribute to pathogenesis include other co-genetic factors (genome wide association studies have identified several markers), wheat-related factors (age of ingestion, type and quantity of wheat) and the way gluten is metabolized in the intestine (Van De Wal et al., 1998; Kagnoff, 2007; Verdu et al., 2015). About 30–40% of the gluten protein consists of glutamine and proline. Since humans are unable to enzymatically break the molecular bonds between these two amino-acids, many immunogenic peptides are produced (Jabri and Sollid, 2006). There remains a possibility that enzymes secreted by the small intestinal microbiota convert some of these immunogenic peptides to non-immunogenic peptides.

While 20–30% of individuals in many countries including India are genetic susceptibility to develop CeD and the majority of them are exposed to wheat, only 1% of them develop CeD. This brings forth the role of other factors such as the gut microbiota in the pathogenesis of CeD (Sanz et al., 2011; Sánchez et al., 2012). Recently, numerous studies have highlighted the potential role of gut microbiota in inflammatory gastrointestinal diseases (Png et al., 2010; Fernandez-Feo et al., 2013; de Sousa Moraes et al., 2014; Schneeberger et al., 2015; Rivière et al., 2016; Zeng et al., 2017).

However, whether the changes in the microbial community structure and function in patients with CeD are cause or effect of the disease state remains unclear to date. In order to answer this question, one has to examine the status of the gut microbiota in the pre-disease state. Recently two studies investigated the microbiota of at-risk children who developed CeD few years after birth. One study observed an increase in *Bifidobacterium breve* and *Enterococcus* spp. in infants that developed active CeD (Olivares et al., 2018). Another study, did not observe any association between microbiota composition and development of CeD during the age of 9 and 12 months (Rintala et al., 2018). Nevertheless, potential microbiota related triggers for development of CeD in later adult life still remain unclear. While 70–80% percent of first-degree relatives (FDRs) of patients with CeD have HLADQ2/DQ8 haplotype (compared to 30% in the general population); only approximately 8.5% of FDRs develop CeD (Singh et al., 2015). Thus, the question arises; why do only few FDRs develop CeD and what is the role of the gut microbiome in disease protection? Indirect evidence of altered microbiota in relatives of patients with CeD is suggested by significantly lower levels of acetic acid and total short chain fatty acids (SCFA), and higher fecal tryptic activity (Tjellström et al., 2007). There is a lack of information regarding the gut microbial composition and function in adult FDRs of patients with CeD. Additionally, it is important to explore the status of the microbiota in both the small intestine, the site of the disease, and feces, as representative of whole gut microbiome. To test the hypothesis that gut microbiome of FDR is different from CeD and could potentially play an important role in the pathogenesis of CeD, we explored the composition of both small intestinal and the whole gut microbiome using Illumina MiSeq in a subset of patients with CeD, FDR and controls. We further investigated the potential microbial functions that are characteristic of FDR and CeD microbiota.

## Materials and Methods

### Human Subjects, Duodenal Biopsies and Fecal Sample Collection

A total of 62 subjects participated in this study including 23 treatment naïve patients with CeD [all HLA-DQ2/DQ8+, having high titre of anti-tissue transglutaminase antibodies (tTG Ab) and having villous abnormalities of modified Marsh grade 2 or more], 15 healthy FDRs of patients with CeD [having normal titre of anti-tTG Ab and having no villous abnormalities of modified Marsh grade 0 or 1], and 24 controls (patients with Hepatitis B Virus carriers or those having functional dyspepsia; having normal titre of anti-tTG Ab and having no villous abnormalities; Table 1). Duodenal biopsies and fecal samples were collected from each of the above mentioned subjects at All India Institute of Medical Sciences, New Delhi, and sent to National Centre for Cell Sciences, Pune for microbiome analysis. The ethics committees of All India Institute of Medical Sciences, New Delhi, and National Centre for Cell Sciences, Pune, India approved the study. Informed and written consent was obtained from all the participants. There was a significant different in the age between the three groups (*p* < 0.05, Kruskal-Wallis test). Further details of patients and controls have been provided in the (Supplementary Table S3).

**Table 1:** Demographic characteristics on study subjects.

Groups	No. of subjects	Age (mean ± S.D.)	Gender^∗^		Sampling site		Villous abnormalities (as per Modified Marsh criteria)					HLA Haplotype			tTG Titre (mean ± SD)
							
			M	F	S	B	0	1	3a	3b	3c	DQ2	DQ8	DQ2+ DQ8+	
CeD	23	23.4 ± 9.5	10	13	21	16	0	0	2	7	14	22	1	0	199.9 ± 72.1
FDR	15	31.6 ± 10.8	6	9	15	13	15	0	0	0	0	13	0	2	4.36 ± 2.6
DC	24	30.6 ± 12.3	22	2	23	14	22	2	0	0	0	6	0	0	4.09 ± 2.8


*M = male, F = female, tTG = Tissue transglutaminase, S = Stool samples, B = Biopsy sample. ^∗^For the DC group our samples are biased by the higher representation of males compared to females.*

### DNA Extraction and 16S rRNA Gene Sequencing

Total DNA was extracted from duodenal biopsies using QIAGEN DNeasy Blood and Tissue kit (QIAGEN, Germany) and fecal samples using the QIAamp fast DNA stool Mini Kit (QIAGEN, Germany) according to the manufacturer’s instructions. We used Illumina MiSeq sequencing to determine the microbial composition of the duodenal biopsies and fecal samples. PCR was set up in 50 μl reaction using AmpliTaq Gold PCR Master Mix (Life Technologies, United States) and with 16S rRNA V4 variable region-specific bacterial primers 515F (5′-GTGCCAGCMGCCGCGGTAA-3′) and 806R (5′- GGACTACHVGGGTWTCTAAT-3′) (Walters et al., 2016).

### Sequence Processing and Bacterial Community Analysis

Illumina Miseq platform rendered a total of 76058052 raw 16S rRNA sequence reads for the 102 fecal and biopsy samples of the diagnosis groups, with an average of 745667 ± 194667 reads per sample. Adapter sequences were trimmed by using Cutadapt (1.18) tool (Martin, 2011) and trimmed reads were pooled as Fasta.gz file format for further analysis in DADA2 (v 1.6.0) pipeline (Callahan et al., 2016). In the first step reads were inspected for read quality profile, the read quality score was decreased (< 30) after 240 bases for forward read and 160 bases for reverse reads. We truncated the forward reads at position 240 (trimming the last 10 nucleotides) and reverse reads at position 160 (trimming the last 90 nucleotide). After quality filtering and removal of bases with a total of 70502947 (92.69%) high-quality reads of the 16S rRNA amplicons were obtained, with an average 691205 ± 181263 reads per sample, ranging from 325350 to 1207169 among samples (Supplementary Table S4). Finally, taxonomic assignment was done by the naive Bayesian classifier method with default setting as implemented in DADA2, against Human Intestinal 16S rRNA gene reference taxonomy database (HITdb v 1.00). Briefly, HITdb is a 16S rRNA gene database based on high-quality sequences specific for human intestinal microbiota, this database provides improved taxonomic up to the species level (Ritari et al., 2015). Unassigned chimeric and sequences of chloroplast and mitochondria were excluded from downstream analysis. Taxonomic assignment successfully mapped 6567144 ASVs, with an average of 64383 ± 29929 ASVs per sample. Finally, from these ASVs, ASV table was constructed and the ASVs generated by the contaminants were removed by using decontam software (Davis et al., 2018) and the output ASV table was used for downstream analyses.

Microbial diversity and composition analysis were done using the R-package phyloseq (v1.22.3) (McMurdie and Holmes, 2013) and microbiome R package (v1.0.2).^1^ To test for similarities in microbial communities between sample types and diagnosis groups Analysis of similarities (ANOSIM) on Bray-Curtis distances was used. ANOSIM is a function in vegan package (v 2.4-4) to calculate significance of PCoA clustering based on the Bray-Curtis distances (Oksanen et al., 2011).

To identify differentially abundant ASVs in pairwise comparisons between diagnosis groups we used DESeq2 (v1.18.0) (Love et al., 2014). All ASVs that were significantly (alpha = 0.01) different in abundance between the diagnosis groups were reported and were adjusted for multiple comparisons using the Benjamini-Hochberg, false discovery rate procedure. Data was visualized using ggplot2 (v 2.2.1) in R (Wickham, 2011, 2017).

### Analysis of 16S rRNA Gene Copies of *H. pylori*

Available full-length 16S rRNA gene sequences of *H. pylori* were downloaded from LPSN-list of prokaryotic names with standing in nomenclature (Parte, 2013). To identify and extract the V4 regions from these 16S rRNA gene sequences, V4 variable region-specific primers 515F (5′-GTGCCAGCMGCCGCGGTAA-3′) and 806R (5′-GGACTACHVGGGTWTCTAAT-3′) were used. Next, to highlight possible similarities and differences between the V4 regions we performed multiple sequence alignment (MSA) using CLUSTAL W 2.0.11 (Larkin et al., 2007). The full-length 16S rRNA gene sequence of *E. coli* strain U 5/41 was used as a reference sequence (Supplementary Information).

### Metagenomic Imputation

Piphillin tool was used to infer metagenome from 16S rRNA ASV counts table and representative sequence of each ASV. Briefly, this tool predicts metagenomes with high accuracy by leveraging the most-current genome reference databases (Iwai et al., 2016). It uses direct nearest-neighbor matching between 16S rRNA amplicons and genomes to predict the represented genomes. Latest version (May, 2017) of KEGG database and 97% of the identity cutoff was selected for the prediction. The output from Piphillin was further analyzed by STAMP statistical tool, ANOVA with *post hoc* Tukey-kramer test was used to identify statistically different KEGG orthologies between diagnosis groups (Parks et al., 2014).

## Results

### Comparison of Fecal and Duodenal Microbial Community in the Study Cohort

The characteristics of the study subjects have been summarized in the Table 1. All the participants were on staple gluten containing diet during sampling for this particular study. After diagnosis of CeD the patients underwent therapy with dietary recommendation to avoid gluten in daily diet. However, in the present study, we do not include samples after dietary changes. The duodenal biopsies and fecal samples were included to investigate differences in both site-specific and whole gut microbial diversity and community structure in patients with CeD, FDRs and DC (non-celiac Disease-Control group). Irrespective of the diagnosis group i.e., CeD, FDR, or DC, the microbial community in the fecal samples was different when compared to the microbial community in duodenal biopsies (ANOSIM statistic R: 0.4998, Significance: 0.001), (Supplementary Figure S1A).

However, there was no significant difference in alpha diversity between the sampling sites (Supplementary Figure S1B).

### Site Specific Comparison of Duodenal and Fecal Microbiota of FDRs, CeD and Control Group

To investigate if patients with CeD, FDRs or DC had site specific dissimilarities in microbiota composition, further analysis was divided based on the sampling site.

**FIGURE 1:**
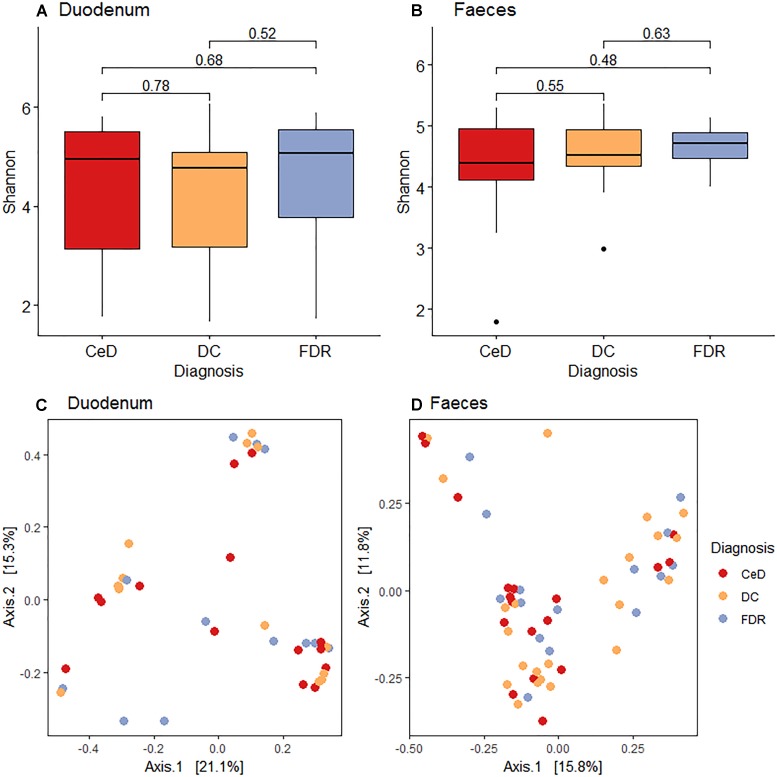
**(A)** Pairwise comparison of alpha diversity of the duodenal microbiota of diagnosis groups. **(B)** Pairwise comparison of alpha diversity of fecal microbiota of diagnosis groups. **(C)** Principle coordinates analysis of microbial community based on Bray-Curtis distance between diagnosis groups in duodenal microbiota. **(D)** Principle coordinates analysis of microbial community based on Bray-Curtis distance between diagnosis groups in fecal microbiota.

Pairwise comparison of alpha diversity of the duodenal microbiota of FDRs, CeD and controls showed no significant difference (Figure 1A). Similarly, for fecal microbiota, no significant difference was observed in the alpha diversity between the diagnosis groups (Figure 1B).

Unconstrained ordination using the Bray-Curtis dissimilarity showed no significant differences in the duodenal microbiota of CeD, FDRs and DC (Analysis of similarities; ANOSIM test; R-statistic = 0.0014, *p* = 0.427) (Figure 1C). Similarly, there was no significant difference in the fecal microbiota of the three diagnosis groups (Analysis of similarities; ANOSIM test; R-statistic = 0.051, *p* = 0.058) (Figure 1D).

### Comparison of Duodenal Microbiota Composition Between the Diagnosis Groups

**FIGURE 2:**
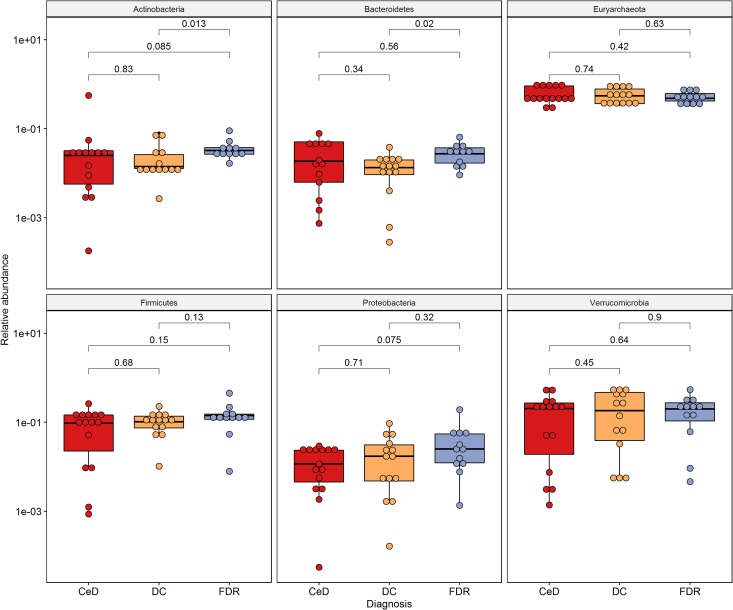
Phylum level distribution of ASVs in duodenal microbiota. Pairwise comparisons were done using Wilcoxon tests.

At phylum level, Actinobacteria, Bacteroides, Euryarchaeota, Firmicutes and Proteobacteria were the dominant members in the duodenal microbiota (Figure 2).

0 The phyla, Actinobacteria (*p* = 0.013) and Bacteroides (*p* = 0.02) were significantly higher in pre-disease state (FDR) in comparison to the DC microbiota. Although at the phylum level there was no significant difference in the abundance of Firmicutes, the order Clostridiales within this phylum showed significantly higher abundance in FDR microbiota compared to DC microbiota (*p* < 0.05) (Supplementary Figure S2). There was no significant difference in abundance of the other major phyla between the FDR and CeD as well as the CeD and DC microbiota (Figure 2).

### Differences in ASV Abundances in Duodenal Microbiota of the Diagnosis Groups

**FIGURE 3:**
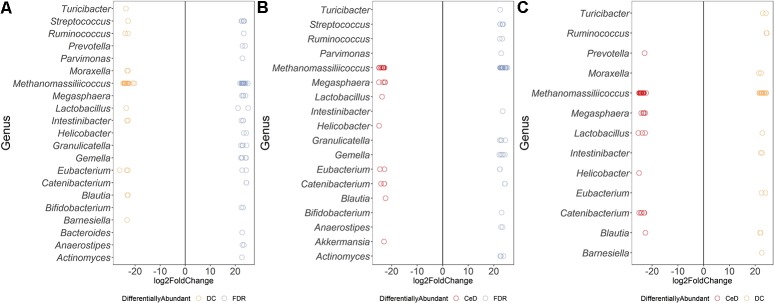
Comparison of differential abundance of microbial ASVs between the diagnosis groups in duodenal microbiota. **(A)** Differential abundance DC vs FDR. **(B)** Differential abundance CeD vs DC **(C)** Differential abundance CeD vs FDR. Only ASVs with significant differences (*P* 0.01) in log2 fold change are depicted.

Differential abundance analysis of ASVs using DESeq2 revealed more than 20-fold higher abundance of ASVs belonging to genera *Parvimonas, Granulicatella, Gemella, Bifidobacterium, Anaerostipes*, and *Actinomyces* in the FDR group compared to both CeD and DC groups (*p*-value < 0.01, Figure 3A,C). In the CeD group, ASVs belonging to genera *Helicobacter* and *Megasphaera* were highly abundant compared to both FDR and DC group (Figure 3B,C).

Investigation of the differentially abundant ASVs between the FDR and CeD group revealed differences in the abundances of specific ASVs within the same genus (Figure 3C). Some ASVs within the archaeal genus *Methanomassiliicoccus* were highly abundant in the CeD group while others were highly abundant in FDR group (Figure 3C). Notably, there is only one species of this archaeal genus, *Methanomassiliicoccus luminyensis*, validly described in literature with a single copy of 16S rRNA gene in its genome (Dridi et al., 2012; Gorlas et al., 2012). This suggests a possibility of either species- or strain- level differences in *Methanomassiliicoccus* between the CeD and FDR which requires further investigation. Additionally, ASVs belonging to genera *Eubacterium* and *Catenibacterium* showed ASV level variation in abundance between the CeD and FDR duodenal microbiota (Figure 3C).

Comparison of both the FDR and CeD duodenal microbiota with that of the DC duodenal microbiota revealed similarities in the abundances of specific ASVs. Both the CeD and FDR duodenal microbiota were characterized by the abundance of ASVs belonging to genus *Prevotella, Megasphaera, Helicobacter* and *Catenibacterium* when compared to the DC duodenal microbiota (Figure 3A,B).

We further checked for similarities and differences between these *Helicobacter* ASVs because, the ASVs of *Helicobacter* were abundant in FDR and CeD duodenal microbiota when compared to DC duodenal microbiota and one specific ASV was abundant in CeD compared to FDR duodenal microbiota. We observed that the ASV identified as differentially abundant in the CeD duodenal microbiota when compared to both FDR and DC microbiota was ASV1811. Contrary to this, ASV2016 and ASV4095 belonging to *H. pylori* were high in abundance in the FDR duodenal microbiota compared to DC duodenal microbiota (Supplementary Information). In view of intra-genomic differences in 16S rRNA gene, we compared the 16S rRNA gene copies of *H. pylori* in publically available genomes. We observed that on average the *H. pylori* genome has two copies of 16S rRNA gene and we did not observe differences between the two copies within a single genome in the V4 region investigated here (Supplementary Information text page 3–7). The analysis used for microbial profiling in the present study employs a well-established algorithm to identify finer sequence level variation and differentiate single nucleotide level difference in the 16S rRNA gene amplicon (Callahan et al., 2016). We suggest that future investigation of CeD and FDR duodenal microbiota need to focus on strain level variations and functional aspects of *H. pylori* using metagenomics and functional omics.

Both CeD and FDR duodenal microbiota were characterized by low abundance (20-fold) of *Barnesiella* when compared to that in DC (Figure 3A,B). Interestingly, the genus *Lactobacillus* showed variation in abundances at ASV level between the three diagnosis groups (Figure 3A–C). Strains of *Lactobacillus* are reported to have probiotic effects, ability to degrade gluten and are often associated with health benefits (Lorenzo Pisarello et al., 2014; Orlando et al., 2014). The differences at ASV level observed in our study indicate the need to investigate the potential impact of species- and/or strain-level differences within the genus *Lactobacillus* in CeD and FDR subjects.

### Comparison of Fecal Microbiota Composition Between the Diagnosis Groups

**FIGURE 4:**
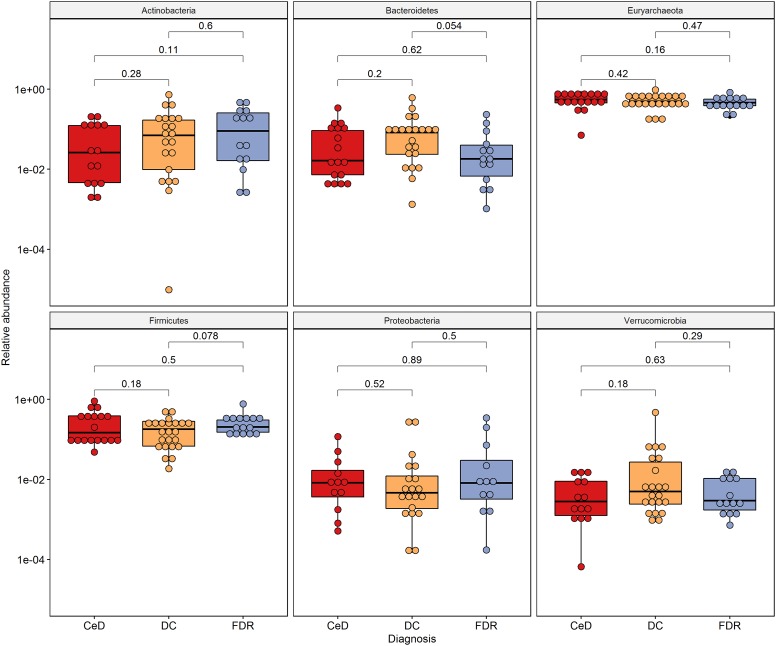
Phylum level distribution of ASVs in fecal microbiota. Pairwise comparisons were done using Wilcoxon tests.

There was no significant difference in abundance of the phyla Proteobacteria, Actinobacteria, Euryarchaeota and Firmicutes (Figure 4) between the diagnosis groups. The phylum Bacteroidetes was found to be marginally lower in abundance in the FDR group when compared to the DC group (*p* = 0.054). Similar trend was observed for order Bacteroidales (*p* = 0.054, Supplementary Figure S3). The order Clostridiales was significantly abundant in FDRs in comparison to DC group (*p* = 0.017) (Supplementary Figure S3). The order Clostridiales was also observed to abundant in duodenal microbiota of FDRs (Supplementary Figure S2).

### Differences in ASV Abundances in Fecal Microbiota of the Diagnosis Groups

When compared to the duodenal microbiota, fewer ASVs were differentially abundant between the diagnosis groups which suggests a lower variation in fecal microbiota at ASV-level.

**FIGURE 5:**
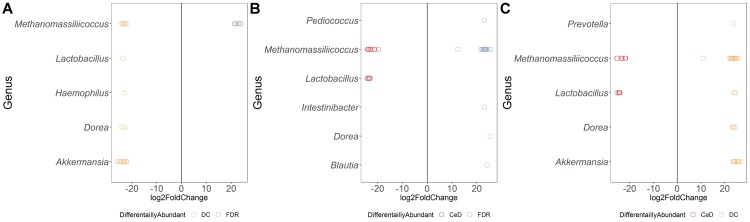
Comparison of differential abundance of microbial ASVs between the diagnosis groups in fecal microbiota. **(A)** Differential abundance DC vs FDR. **(B)** Differential abundance CeD vs FDR. **(C)** Differential abundance CeD vs DC. Only ASVs with significant differences (*P* < 0.01) in average log2 fold change are depicted.

Both FDR and CeD fecal microbiota was characterized by a 20-fold decrease in abundance of ASVs belonging to genera *Dorea* and *Akkermansia* (Figure 5A,B). In addition, when compared to DC, FDR fecal microbiota showed lower abundance of ASVs belonging to *Lactobacillus* and *Haemophilus* while, fecal microbiota of CeD had lower abundance of *Prevotella* (Figure 5A,B). Interestingly, CeD fecal microbiota had ASV level differences in abundance of *Lactobacillus* when compared to DC fecal microbiota (*p* < 0.01, Figure 5B). Similarly, *Methanomassiliicoccus* showed ASV level variation in abundances between the three diagnosis groups (*p* < 0.01, Figure 5A–C).

Comparison of ASV abundances between the FDR and CeD fecal microbiota revealed higher abundance of ASVs from genera *Pediococcus, Intestinibacter, Blautia* and *Dorea* in the FDR microbiota (*p* < 0.01) (Figure 5C). However, in comparison with DC fecal microbiota, FDR microbiota had 20-fold lower abundance of ASVs related to *Akkermansia* and *Dorea* (*p* < 0.01).

### Imputed Metagenome of FDR and CeD Fecal Microbiome Shows Reduced Proportion of Genes Involved in Gluten Metabolism

**FIGURE 6:**
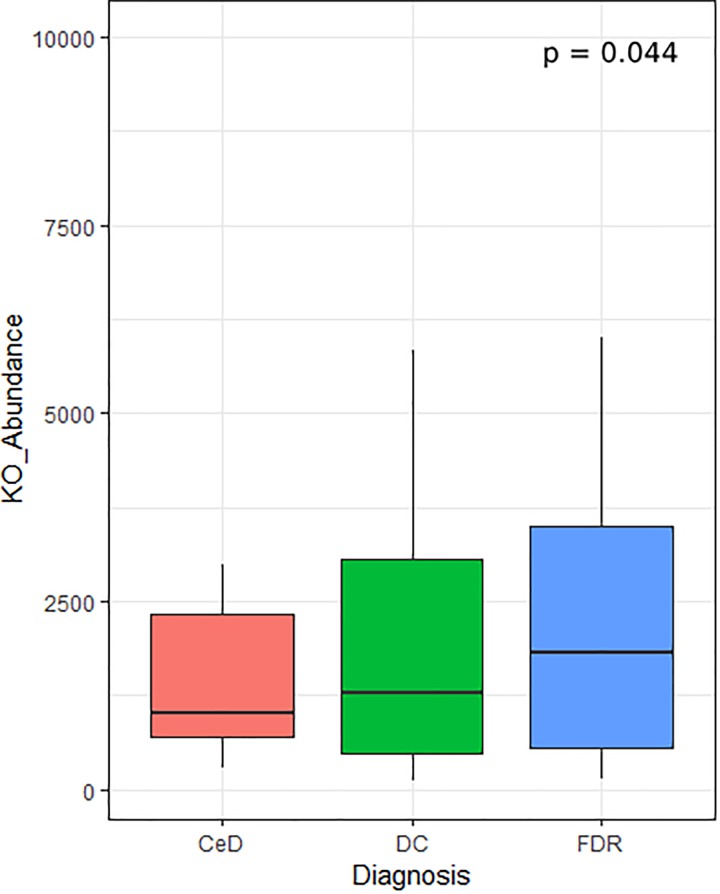
KO abundance for Xaa-pro dipeptidase (K01271) enzyme in feces inferred from predicted metagenome for fecal samples. Comparison was done using ANOVA.

In addition to differentially abundant microbial ASVs, different study groups might have altered metabolic potential. Of specific interest were the enzymes related to glutenases as they play a role in breakdown of gliadin residues. We followed Piphillin workflow to predict functional profile of fecal and duodenal microbiota (Iwai et al., 2016). A total of 159 KEGG orthologies (KO) were significantly different between diagnosis groups in the fecal microbiota (Supplementary Table S1). Among these the KO abundance for Xaa-pro dipeptidase (K01271, Prolidase) enzyme which is known to have role in gluten degradation was found to be significantly reduced in CeD as compared to FDR and DC fecal microbiota (Figure 6). Notably, we did not observe any significant difference in the predicted abundance of prolidase in the duodenal microbiota (Supplementary Table S2).

## Discussion

The aim of the present study was to investigate differences in the duodenal and fecal microbiota of pre-diseased state i.e., FDRs subjects compared to diseased state i.e., CeD and DC. The FDR group was included for two main reasons: (1) They represent a population which is genetically-susceptible to develop CeD; (2) They provide a unique opportunity to identify features of the host as well as of the associated microbiota that may be involved in the protection against developing CeD. We collected both duodenal biopsies and fecal samples to investigate both local and overall changes in the microbiota in FDR, patients with CeD and DC. To the best of our knowledge, reports on site specific microbiota patterns in adult patients with CeD remain scarce, and no results on both site specific and whole gut microbiome on FDRs have been reported to date. Present study provides an overall view on differences of both site-specific changes as well as changes in the fecal microbiota of FDRs, CeD and DC.

The duodenal microbiota of CeD was characterized by high abundance of *Helicobacter* and *Megasphaera* compared to both FDR and DC group. In addition, comparison of *Helicobacter* ASVs suggested ASV-level variation between FDR and CeD duodenal microbiota. Previously, CeD patients with *H. pylori* gastritis were reported to have an increased number of intraepithelial lymphocytes in the duodenal mucosa (Villanacci et al., 2006). On the contrary, there are also reports which have failed to reveal a relationship between *H. pylori* and CeD and found that *H. pylori* presence was inversely associated with CeD (Lebwohl et al., 2013). In the present study, the ASV which is abundant in CeD is different from those ASVs which are enriched in FDR, emphasizing the need for investigating strain level variations in *H. pylori* and its potential impact on pathophysiology of CeD. There were other genera which showed ASV level difference between the microbiota of the CeD, FDR and DC such as, *Streptococcus, Ruminococcus, Methanomassiliicoccus, Catenibacterium, Intestinibacter*, and *Blautia*. Specific interest would be for identifying those ASVs that are differentially abundant between the FDR and CeD microbiota. In this comparison between the pre-disease and disease state, we observed a reduced abundance of ASVs related to *Ruminococcus, Parvimonas, Intestinibacter, Granulicatella, Gemella, Bifidobacterium, Anaerostipes*, and *Actinomyces* in the diseased state. The species from genera *Ruminococcus* and *Anaerostipes* are known to produce SCFA such as acetate and butyrate that have beneficial effect on the host (Ze et al., 2012; Morrison and Preston, 2016; Shetty et al., 2017). The genus *Granulicatella* is commonly associated with diseases such as cancer, Crohn’s disease and bacteremia (Woo et al., 2003; Nakatsu et al., 2015; Qiu et al., 2017). The bacterial species within the genera *Actinomyces, Streptococcus, Bifidobacterium*, and *Anaerostipes* are known to possess gluten degrading enzymes, probiotic properties and ability to produce SCFA, respectively (Barrangou et al., 2009; Fernandez-Feo et al., 2013, Couvigny et al., 2015; Morrison and Preston, 2016, Rivière et al., 2016). Moreover, a strain belonging to *Bifidobacterium* was recently reported to prevent gluten-related immunopathology in mice (McCarville et al., 2017). Higher abundances of ASVs belonging to the above-mentioned genera in small intestine of FDRs compared to CeD may indicate their potential protective role in pre-disease state.

In comparison to duodenal biopsies, a smaller number of ASVs were differentially abundant between the diagnosis groups in fecal samples. This indicates more disrupted microbiome at disease site than faecal gut microbiome and highlights the importance of inclusion of biopsy samples in present study. We observed ASV level variation in the both fecal and duodenal microbiota between the diagnosis groups. Previously, a higher abundance of *Lactobacillus* was observed in the oral microbiome of patients with CeD (Tian et al., 2017). Moreover, there are reports stating that the certain *Lactobacillus* species degrade gliadin and increases the availability of antigenic peptides (Engström et al., 2015). In the present study, higher abundance of different ASVs of *Lactobacillus* in CeD and in FDR microbiota may suggest strain level differences in ability to breakdown gluten into either pro-inflammatory or anti-inflammatory peptides in the small intestine. However, this will require more in-depth characterization of the strain-level variation in functional capabilities of *Lactobacillus* species. Another important observation from differential abundance analysis was the significantly lower abundance of *Barnesiella* in CeD and FDR compared to DC duodenal microbiota and significantly lower abundance of *Akkermansia* in fecal microbiota of both CeD and FDR compared to DC. While *Akkermansia* was highly abundant in CeD microbiota when compared to FDR microbiota. Both of these genera are known to degrade mucus and produce SCFAs which in turn strengthens the health of enterocytes and inhibits intestinal inflammation (Desai et al., 2016; Ravcheev and Thiele, 2017). Therefore, there is a need for more detailed investagion of these bacteria commonly residing in the mucus layer.

Through metagenome prediction method, we found that the gene abundance for Xaa-pro Dipeptidase enzymes was less in CeD as compared to FDR and DC microbiota. This enzyme shows a high specificity for proline residues present in gluten and hydrolyze the peptide bond (Park et al., 2004). These observations suggest that the FDR and CeD fecal microbiota differs in the bacterial composition and that there is a difference in specific bacteria that are capable of gluten degradation. As a consequence, this may impact gluten processing and the presentation of immunogenic gluten epitopes to the immune system. However, the observations of the predicted metagenome have to be validated with *in vitro* enzyme assay.

Overall, we observe differences at ASV level between the FDR and CeD microbiota. We do not observe major differences in community diversity and structure from both alpha diversity and community dissimilarity analysis. The potential species and/or strain level variations and functional aspects identified in this study emphasize the need for well-designed mechanistic follow-up studies using bacteria identified as different between the disease and pre-disease states.

However, metagenomic studies of biopsy samples remain a challenge because of high proportion of host DNA. Thus, predictive metagenomics using 16S rRNA gene as a practical solution was employed for biopsies. In this initial exploratory study, we investigated the gut microbiome with respect to the disease status only and future studies considering other confounding factors such as diet, body mass index age, sex, frequency and quantity of gluten intake among others will be required for a better understanding the gut microbiome in CeD and FDRs. Additionally, the control group in our study was not healthy subjects but patients with functional dyspepsia. These subjects were used as proxy since invasive sampling procedures such as endoscopy from clinically healthy subjects is not permitted under the institutional regulations. Since one of the aims of the present study was to identify similarities and differences between the FDR and CeD microbiota, a control group with a different disease was used as comparison group. This procedure of using a control group with a disease other than the focus of the main study is common practice in epidemiological studies (Coggon et al., 2009). However, this could have also resulted in underestimating the number of ASVs that could be variable between FDR vs DC and CeD vs DC, especially in the CeD group because the proposed disease model for functional dyspepsia includes low-grade duodenal inflammation (Talley et al., 2017). Despite the fact that, *H. pylori* is a recognized causative agent of functional dyspepsia, we observe high abundance of ASVs related to *H. pylori* in CeD and FDR microbiota (Sugano et al., 2015). Therefore, the possible role of *H. pylori* in pathophysiology of CeD requires further investigation. Based on the findings of the present study, it can be hypothesized that the microbiota of FDR represents a potentially balanced, non-inflammatory state as compared to that of microbiota of patients with CeD in genetically pre-disposed subjects. High abundance of known pro-inflammatory bacteria such as those related to *Helicobacter* could have a critical role in the pathogenesis of CeD. In addition, expansion of specific species or strains of gluten degrading bacteria which breakdown gluten into pro-inflammatory peptides may have a pivotal role in the pathogenesis of CeD. Exposure to drugs (e.g., proton pump inhibitors) and antibiotics have been hypothesized to select particular strains of bacteria in humans (Domínguez-Bello et al., 2008). A long-term follow-up of FDRs of patients with CeD will be crucial to identify triggers such as dietary changes, lifestyle changes, medications, specifically antibiotics that could affect the microbiota homeostasis in them and factors that lead to transition toward a pro-inflammatory microbiota from a non-inflammatory microbiota.

## Conclusion

Significant differences at ASV level suggest that specific bacteria like *Helicobacter* may be important for pathogenesis of CeD. Higher abundance of potentially beneficial bacterial ASVs especially those belonging to SCFA producing genera in FDRs suggest that there may be a protective role of these in CeD development. Moreover, the predicted differences in gluten metabolism potential by FDR and CeD microbiota point toward the need for investigating functional capabilities of specific bacteria in healthy FDR and CeD patients.

## Data Availability

Sequence data generated in this study is available from the NCBI Sequence Read Archive within the Bioproject ID accession PRJNA385740 (https://www.ncbi.nlm.nih.gov/bioproject/?term~=~PRJNA385740) and to reproduce the analysis done in R, the R Markdown file and required data are available at https://github.com/rahulnccs/Comparison-of-Small-Gut-and-Whole-Gut-Microbiota-of-First-Degree-Relatives-with-Adult-Celiac-Disease.

## Author Contributions

GM, YS, and VA conceived, designed, and supervised the study. GM recruited the patients, and performed the diagnosis and endoscopic examination. GK performed the HLA test. AV, KB, and AM were responsible for storage and maintenance of the collected biological samples (duodenal biopsy/stool). RB and PP extracted the genomic DNA. DB, BS, and RP were involved in amplicon sequencing. SS, DD, and RB performed the bioinformatics analysis for amplicon data. SS, RB, DD and GM acquired and interpreted the data, and drafted the manuscript. YS, DD, and VA critically reviewed the manuscript. All authors have read and approved the final manuscript.

## Conflict of Interest Statement

DB, BS, and RP were employed by company AgriGenome Labs Pvt Ltd. Kerala, India. The remaining authors declare that the research was conducted in the absence of any commercial or financial relationships that could be construed as a potential conflict of interest.

## Footnotes

1 https://github.com/microbiome/microbiome

## Supplementary Material

FIGURE S1 Principal coordinates analysis (PCoA) of the fecal and duodenal microbiota based on Bray–Curtis distance. **(B)** Comparison of alpha diversity measures between sampling sites.

FIGURE S2 Order level distribution of ASVs in duodenal microbiota. Pairwise comparisons were done using Wilcoxon tests.

FIGURE S3 Order level distribution of ASVs in fecal microbiota. Pairwise comparisons were done using Wilcoxon tests.

TABLE S1 Significantly different KEGG orthologies (KO) between diagnosis groups in fecal microbiota. Analyzed using the STAMP statistical tool, ANOVA with *post hoc* Tukey–kramer test was used to identify statistically different KEGG orthologies between diagnosis groups.

TABLE S2 Significantly different KEGG orthologies (KO) between diagnosis groups in duodenal microbiota. Analyzed using the STAMP statistical tool, ANOVA with *post hoc* Tukey–kramer test was used to identify statistically different KEGG orthologies between diagnosis groups.

TABLE S3 Details of the study subjects including disease status, Marsh values, age, gender, and tTg titre.

TABLE S4 Number of sequencing reads per sample at each stage of data analysis is given below.

INFORMATION Differential Abundance of Amplicon Sequence Variants of *Helicobacter.* Multiple sequence alignment was performed by CLUSTAL 2.0.11.

## Abbreviations

ANOSIM: analysis of similarities
ASV: amplicon sequence variant
CeD: celiac disease
DADA2: divisive amplicon denoising algorithm 2
DC: diseased controls (dyspeptic)
FDR: first degree relatives
HLA: human leukocyte antigen
KEGG: kyoto encyclopedia of genes and genomes
PCoA: principal coordinates analysis
rRNA: ribosomal ribonucleic acid
SCFA: short chain fatty acids
tTG: tissue transglutaminase
