# Supplementary figures and images for: Comparison of Small Gut and Whole Gut Microbiota of First-Degree Relatives With Adult Celiac Disease Patients and Controls
Source: Front Microbiol. 2019 Feb 8;10:164. doi: 10.3389/fmicb.2019.00164 (PMC6376745; doi:10.3389/fmicb.2019.00164)

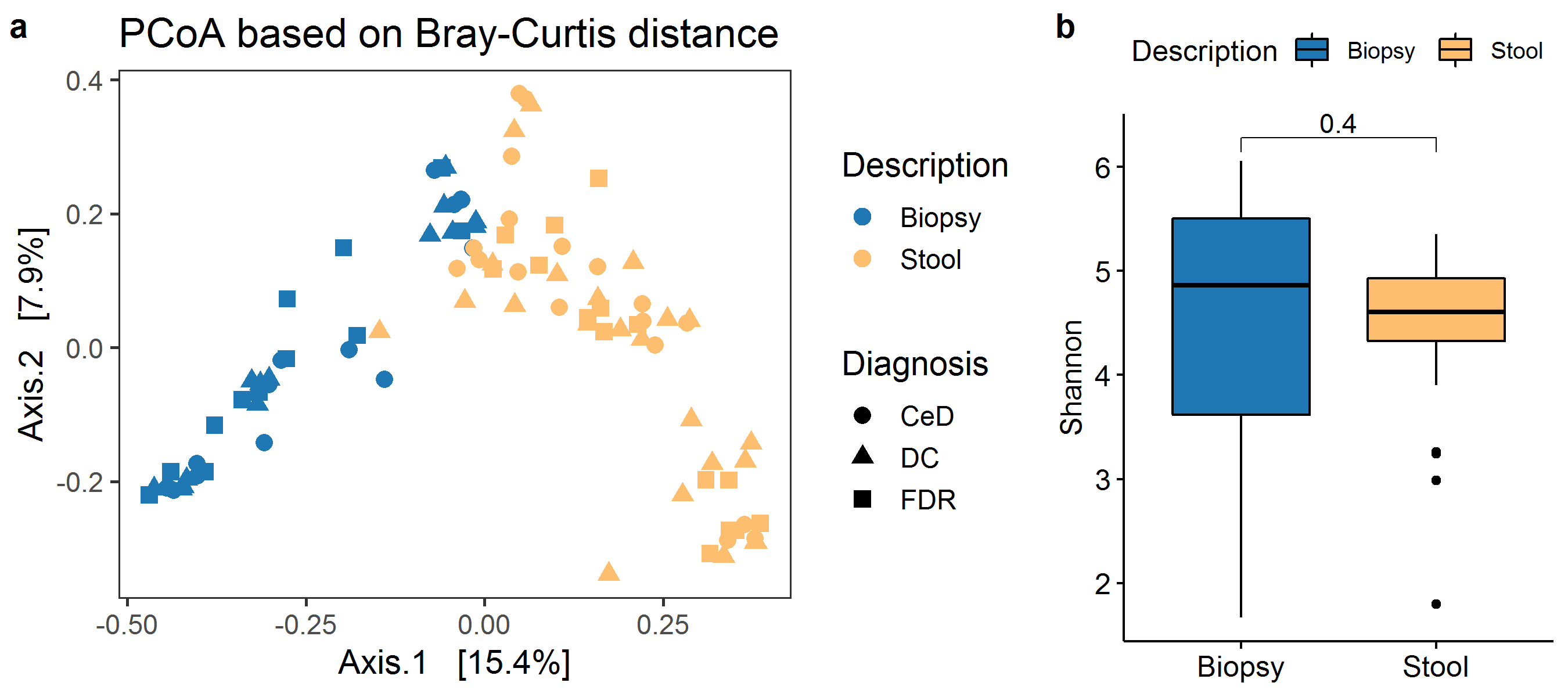

Supplement: FIGURE S1 — Principal coordinates analysis (PCoA) of the fecal and duodenal microbiota based on Bray–Curtis distance. (B) Comparison of alpha diversity measures between sampling sites. [file Image_1.TIFF]

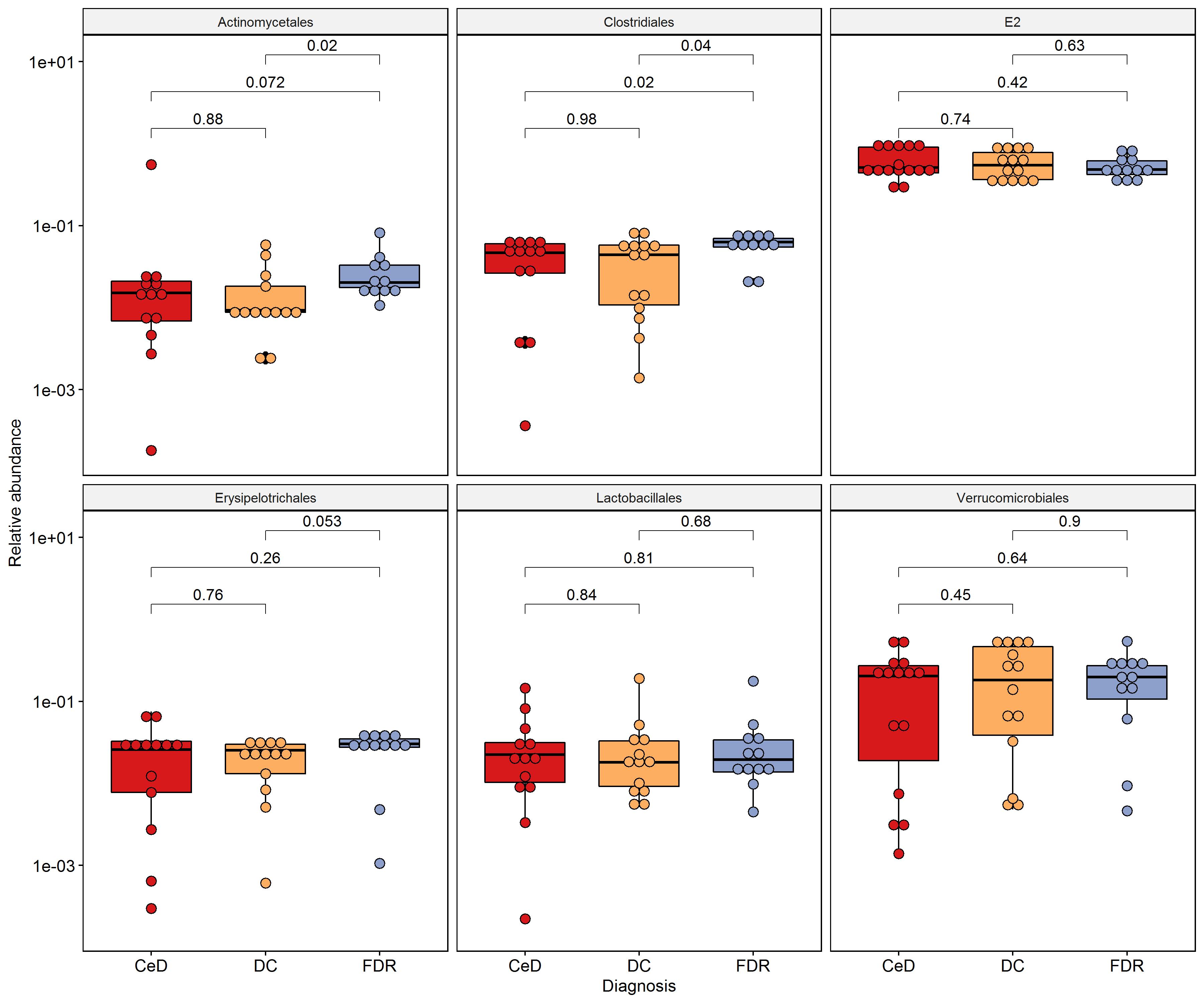

Supplement: FIGURE S2 — Order level distribution of ASVs in duodenal microbiota. Pairwise comparisons were done using Wilcoxon tests. [file Image_2.JPEG]

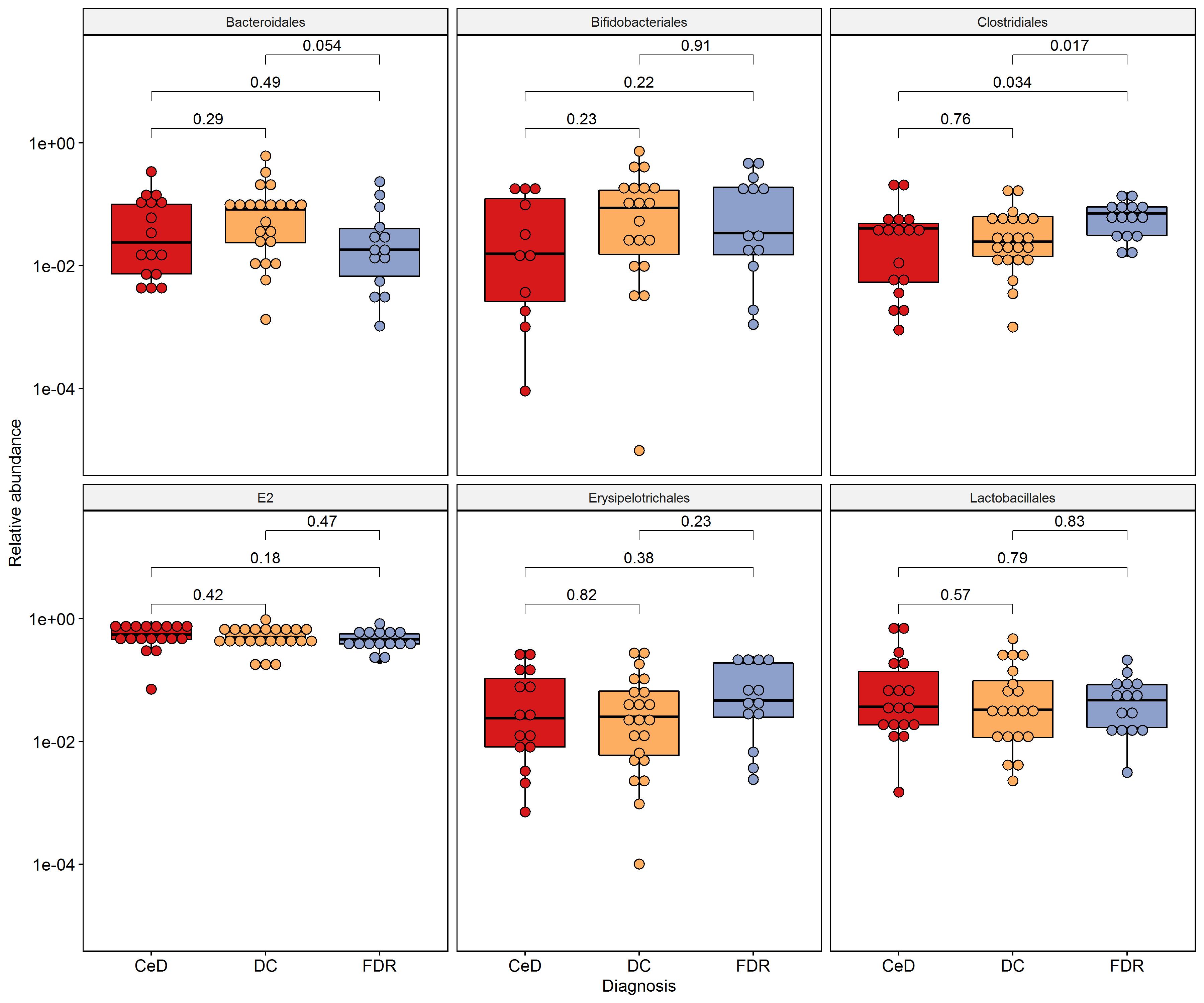

Supplement: FIGURE S3 — Order level distribution of ASVs in fecal microbiota. Pairwise comparisons were done using Wilcoxon tests. [file Image_3.JPEG]
