# Supplementary material for: Comparison of Small Gut and Whole Gut Microbiota of First-Degree Relatives With Adult Celiac Disease Patients and Controls
Source: Front Microbiol. 2019 Feb 8;10:164. doi: 10.3389/fmicb.2019.00164 (PMC6376745; doi:10.3389/fmicb.2019.00164)
Supplement: TABLE S1 — Significantly different KEGG orthologies (KO) between diagnosis groups in fecal microbiota. Analyzed using the STAMP statistical tool, ANOVA with post hoc Tukey–kramer test was used to identify statistically different KEGG orthologies between diagnosis groups. [file Data_Sheet_1.PDF]

Supplementary Table 1: Significantly different KEGG orthologies (KO) between diagnosis groups in fecal microbiota. Analyzed using the STAMP statistical tool, ANOVA with post hoc Tukey-kramer test was used to identify statistically different KEGG orthologies between diagnosis groups.

Significantly Different KOs in Fecal Microbiota KO for xaa-prodipeptidase (K01271) is highlighted

				**CeD**		**DC**		**FDR**	
Sr.No	KO_ID	p-values	Effect size	mean rel. freq. (%)	std. dev. (%)	mean rel. freq. (%)	std. dev. (%)	mean rel. freq. (%)	std. dev. (%)
1	K00148	0.048624236	0.107830699	2.67E-05	7.13E-05	4.23E-05	0.000108197	0.002020984	0.004884429
2	K00243	0.041426366	0.113207998	0.032581387	0.02995869	0.014052691	0.017063542	0.018542685	0.019053503
3	K00265	0.027833898	0.126415982	0.036555176	0.027905501	0.0588903	0.027253957	0.057979359	0.025446339
4	K00282	0.046367726	0.109429058	0.000992932	0.001482554	0.001225705	0.003013767	0.005327552	0.00943617
5	K00297	0.040397915	0.114048859	0.050341162	0.024282975	0.0676248	0.021143364	0.066708966	0.020548641
6	K00533	0.038425094	0.11572114	2.22E-05	7.26E-05	2.79E-05	0.000122641	0.000392977	0.000852435
7	K00571	0.005236012	0.179790961	0.052823538	0.029425672	0.029648196	0.022507283	0.026852955	0.019886709
8	K00687	0.048177214	0.108141589	0.004059438	0.006474274	0.001439402	0.002725633	0.000559765	0.001139924
9	K00752	0.035354157	0.11849624	0.004184935	0.00758878	0.000976563	0.001389267	0.000417999	0.000746169
10	K00784	0.038478883	0.115674459	0.041626941	0.026970869	0.02384446	0.017815274	0.027471658	0.01912013
11	K00805	0.024390612	0.130758446	0.04451074	0.046465559	0.01679353	0.021785667	0.020511487	0.021607406
12	K00857	0.036184223	0.11772393	0.041518386	0.029894876	0.022651587	0.017679569	0.026731499	0.01846707
13	K00863	0.009396042	0.161491717	0.005987968	0.009420054	0.000752816	0.001662931	0.000843533	0.001896501
14	K00867	0.013434627	0.150101613	0.039194593	0.023715318	0.021598924	0.01789125	0.021213697	0.016942646
15	K00876	0.038806988	0.115391072	0.045240084	0.027296611	0.025538221	0.018527107	0.033922714	0.024147903
16	K00917	0.022539316	0.133343857	0.014920235	0.024695404	0.002973195	0.004879086	0.002504777	0.003388053
17	K00991	0.048104272	0.108192581	0.043090428	0.028853838	0.06192992	0.026818461	0.065251076	0.026048022
18	K01179	0.048184335	0.108136615	0.001977187	0.003532169	0.0003306	0.000500648	0.003092383	0.004979879
19	K01209	0.012155684	0.153303967	0.004381455	0.00460138	0.004884184	0.004111393	0.010121278	0.008370385
20	K01215	0.014325619	0.14803967	0.01984983	0.023882328	0.005094053	0.006172616	0.006835151	0.013971344
21	K01220	0.013624175	0.149652163	0.012480517	0.019666231	0.00239396	0.004637787	0.001501602	0.003135685
22	K01261	0.047281994	0.10877262	0.007574172	0.012372021	0.003381034	0.00470298	0.000707367	0.001283061
23	K01267	0.049915267	0.106948032	0.010412225	0.015721588	0.003173572	0.005506816	0.00318778	0.003554251
24	K01271	0.043722634	0.111400839	0.036433186	0.023269561	0.020904136	0.017840219	0.023137189	0.016728709
25	K01488	0.024191137	0.131027771	0.029080713	0.024308587	0.016099777	0.012682274	0.013784948	0.010411367
26	K01575	0.039708534	0.114624108	0.024619141	0.024971654	0.009956265	0.016654746	0.009624949	0.014262097
27	K01577	0.040263597	0.114160195	0.016181865	0.017779461	0.007242092	0.008800465	0.006222635	0.007632635
28	K01610	0.014876237	0.146826268	0.03552262	0.024765026	0.017568596	0.012140144	0.023049908	0.01798539
29	K01619	0.035900148	0.117986302	0.035504032	0.025648276	0.01946971	0.013424693	0.024424895	0.015869586
30	K01666	0.009576427	0.160889801	0.000947701	0.001719545	0.000491813	0.001021226	0.004330493	0.006792235
31	K01679	0.030881728	0.122983817	0.034761739	0.025452589	0.01912676	0.014680427	0.020252554	0.015412772
32	K01754	0.022907848	0.132813288	0.055218097	0.025778492	0.074790537	0.017874977	0.070471644	0.021994176
33	K01841	0.04076128	0.113749443	0.000105744	0.000241557	4.80E-05	0.000104858	0.000453124	0.00086795
34	K01902	0.04954746	0.10719724	0.036373622	0.028897672	0.057741344	0.026375532	0.055643484	0.028539553
35	K01903	0.04954746	0.10719724	0.036373622	0.028897672	0.057741344	0.026375532	0.055643484	0.028539553
36	K01914	0.005330935	0.179234686	0.035290765	0.024892365	0.018560394	0.013139287	0.016878377	0.011303851
37	K01916	0.040296098	0.114133223	0.037403203	0.022720162	0.021395757	0.017686368	0.024717775	0.017826345
38	K01926	0.029451264	0.124552035	0.03094717	0.0277143	0.013607348	0.012919674	0.017350799	0.017945343
39	K01960	0.00192988	0.210108976	0.000588584	0.001195838	0.00037003	0.000640109	0.003870344	0.005447619

				CeD		DC		FDR	
Sr.No	KO_ID	p-values	Effect size	mean rel. freq. (%)	std. dev. (%)	mean rel. freq. (%)	std. dev. (%)	mean rel. freq. (%)	std. dev. (%)
40	K01978	0.010961636	0.156601094	0.020854213	0.019196346	0.009136028	0.010659535	0.007938881	0.006323572
41	K02041	0.010988639	0.156522785	0.025378601	0.023052932	0.011235881	0.010765418	0.01064062	0.009634172
42	K02042	0.016999795	0.142519426	0.039472895	0.044913894	0.01488842	0.014486999	0.015108256	0.014066484
43	K02044	0.022832132	0.132921621	0.033766853	0.041544658	0.013155019	0.011512889	0.011745664	0.010658861
44	K02238	0.040778877	0.113735008	0.051260358	0.030041126	0.034314996	0.025298013	0.028407266	0.021408325
45	K02248	0.026271495	0.128318324	0.021620087	0.025579448	0.007180115	0.014067462	0.007178665	0.008054925
46	K02411	0.010293832	0.158599223	0.003885865	0.004356056	0.005884894	0.006925851	0.01406254	0.015376748
47	K02412	0.011573678	0.154870135	0.003940974	0.004364744	0.006007424	0.007100176	0.01406254	0.015376748
48	K02419	0.013537424	0.149857113	0.004162329	0.00430423	0.006024911	0.007088407	0.014639402	0.016837259
49	K02476	0.026540859	0.127982715	0.004367419	0.008041163	0.000399012	0.000845835	0.0007825	0.001659089
50	K02530	0.023620111	0.131810733	0.013692219	0.020856879	0.003922261	0.007174814	0.001967513	0.003208646
51	K02531	0.024812561	0.130195662	0.010269699	0.019199722	0.001404587	0.00243657	0.000738446	0.001899506
52	K02556	0.037505774	0.116528829	0.004731838	0.005658347	0.006355593	0.007381141	0.014945309	0.019283485
53	K02557	0.011603445	0.154788213	0.005000443	0.006068854	0.006410449	0.007403794	0.016710078	0.018859158
54	K02652	0.046464186	0.109359216	0.025573833	0.029784765	0.052696784	0.038194258	0.047648426	0.03181704
55	K02669	0.028614076	0.125504205	0.038281173	0.027752662	0.063650354	0.032902079	0.058169345	0.024452035
56	K02744	0.027042343	0.12736654	0.013508094	0.016981259	0.00618754	0.008014496	0.002905019	0.003787015
57	K02749	0.018536829	0.139714016	0.017835043	0.017225738	0.008729821	0.009593885	0.005979275	0.007191646
58	K02750	0.018536829	0.139714016	0.017835043	0.017225738	0.008729821	0.009593885	0.005979275	0.007191646
59	K02786	0.013034682	0.151070325	0.007817439	0.012348007	0.001564036	0.003291014	0.000709075	0.001297661
60	K02787	0.009026145	0.16276159	0.00968267	0.014427606	0.001896006	0.003723267	0.001113853	0.002316758
61	K02788	0.009026145	0.16276159	0.00968267	0.014427606	0.001896006	0.003723267	0.001113853	0.002316758
62	K02808	0.027888234	0.126351688	0.042276544	0.048849311	0.017023885	0.02057754	0.015526111	0.016035461
63	K02809	0.017581448	0.141430123	0.054906211	0.057357521	0.022930952	0.023812669	0.022776307	0.0160036
64	K02810	0.017581448	0.141430123	0.054906211	0.057357521	0.022930952	0.023812669	0.022776307	0.0160036
65	K02824	0.035279704	0.118566363	0.038597567	0.024957507	0.022803617	0.018222716	0.022710444	0.016139715
66	K02825	0.028343115	0.12581813	0.052039639	0.049746356	0.025701499	0.02552537	0.022312799	0.020324407
67	K02837	0.025623976	0.129138833	0.042780768	0.025794916	0.024783194	0.018993289	0.026095585	0.018253433
68	K03090	0.016346672	0.143786167	0.000863762	0.001358921	0.000315567	0.000469283	0.004847034	0.008883575
69	K03303	0.026469807	0.128070922	0.021348674	0.025824677	0.007970715	0.010462278	0.00745388	0.007458618
70	K03311	0.00504423	0.180945097	0.069012035	0.056973604	0.029150865	0.021129208	0.034334011	0.027652407
71	K03442	0.040540379	0.11393116	0.042099696	0.024880884	0.023877318	0.017773253	0.029594353	0.023257009
72	K03529	0.034364434	0.119440235	0.037883967	0.029706411	0.01861371	0.019871889	0.020998357	0.019434404
73	K03587	0.028304883	0.125862656	0.03798463	0.029228268	0.061142348	0.025716475	0.059227289	0.028535853
74	K03628	0.028949227	0.125119846	0.051104428	0.027538359	0.075024293	0.029122823	0.066818958	0.022575518
75	K03652	0.037333521	0.116682283	0.021711845	0.028029401	0.00679885	0.008886161	0.009609663	0.01211935
76	K03693	0.031312136	0.122525628	0.014647419	0.021963062	0.003115913	0.004765172	0.004876757	0.009195714
77	K03710	0.04846844	0.107938737	0.064700207	0.071077843	0.028763547	0.032797851	0.031020808	0.024061915
78	K03763	0.045792995	0.109848116	0.033432482	0.031094859	0.01462795	0.017482263	0.019744016	0.018934991
79	K03785	0.021051739	0.135573947	0.046942013	0.032246049	0.029809919	0.021379918	0.022565431	0.018136766
80	K03930	0.034283425	0.119518656	0.005768213	0.008932047	0.00172654	0.00290144	0.001174221	0.001249677

				CeD		DC		FDR	
Sr.No	KO_ID	p-values	Effect size	mean rel. freq. (%)	std. dev. (%)	mean rel. freq. (%)	std. dev. (%)	mean rel. freq. (%)	std. dev. (%)
81	K04061	0.030064657	0.123870788	0.000825325	0.001200451	0.000383002	0.00079275	0.003380119	0.006238761
82	K04074	0.03918669	0.115065983	0.029889755	0.028125084	0.012663432	0.016731283	0.016125914	0.015890236
83	K04094	0.037793258	0.116274225	0.024216709	0.026053197	0.009167178	0.00924435	0.013281861	0.015741691
84	K04516	0.015501125	0.145500486	0.01301876	0.01894082	0.002726612	0.003427787	0.003448851	0.00567656
85	K05896	0.037724774	0.116334707	0.035884577	0.02860982	0.016953307	0.019214805	0.021076634	0.019539045
86	K05985	0.045884553	0.109781021	0.029968622	0.027942302	0.013377346	0.016846035	0.015987306	0.016132888
87	K06024	0.043183895	0.111816482	0.037334075	0.02990542	0.018618338	0.019948644	0.021095175	0.019524377
88	K06208	0.018891202	0.13909904	0.000231172	0.000517703	7.96E-05	0.000268956	0.004476553	0.009199769
89	K06320	0.036035537	0.117861009	0.000489776	0.000869127	0.000234272	0.000583303	0.00173158	0.003068264
90	K06726	0.044058597	0.111144128	0.031205538	0.022178238	0.01736192	0.017813001	0.017224803	0.013457588
91	K06878	0.027422491	0.126906735	0.034205732	0.023023612	0.018744277	0.017358916	0.018315564	0.016471804
92	K06915	0.020036002	0.137185572	0.019418603	0.015866229	0.010104365	0.01151265	0.007724297	0.007396338
93	K06928	0.046093128	0.10962865	0.000203849	0.000593628	0.001572441	0.003104899	5.65E-05	0.000121926
94	K06967	0.039088255	0.115149968	0.032214046	0.030087454	0.013696239	0.017152767	0.017866624	0.018016135
95	K06994	0.029241378	0.124788277	0.022175834	0.029007194	0.005128432	0.007084535	0.011261773	0.017169618
96	K07005	0.014137092	0.148465463	0.017214578	0.015166224	0.031918051	0.019666834	0.031002875	0.009722035
97	K07010	0.031262362	0.122578304	0.036594303	0.04278545	0.012588505	0.014157844	0.017389649	0.021102963
98	K07029	0.040235516	0.114183517	0.02940264	0.027244889	0.012635689	0.016584603	0.016178686	0.015442761
99	K07030	0.028169652	0.126020617	0.032883713	0.028874615	0.014173547	0.016854247	0.0179354	0.017289514
100	K07104	0.047855439	0.108367096	0.006560515	0.00852493	0.001101838	0.001379764	0.003877179	0.008704941
101	K07118	0.018137214	0.140421227	0.023576813	0.026289878	0.007617025	0.010689397	0.010071426	0.011743367
102	K07305	0.033075111	0.120710018	0.036413383	0.023103289	0.021093574	0.019188773	0.020263274	0.015819177
103	K07321	0.021873142	0.134324476	0.00047825	0.001254655	0.000446137	0.000822984	0.002349487	0.003730717
104	K07334	0.043325596	0.111706677	0.009760128	0.017694301	0.001910967	0.002709565	0.001919937	0.003407472
105	K07335	0.043604343	0.111491678	0.02953122	0.035035997	0.011196832	0.012732899	0.013667507	0.016226374
106	K07455	0.049327904	0.10734685	0.012388246	0.013975954	0.020925553	0.015260811	0.025070717	0.013993629
107	K07723	0.005838282	0.176414163	0.000960823	0.001375985	0.000349523	0.000719202	0.008066307	0.013701656
108	K07742	0.032730525	0.12105745	0.03527708	0.028509047	0.01641377	0.017780272	0.020192128	0.019668501
109	K08138	0.049683461	0.107104886	0.000490848	0.000957558	0.000159297	0.000311844	0.002299093	0.004862247
110	K08152	0.040130043	0.114271252	0.005309295	0.008874149	0.000417562	0.001078426	0.00240032	0.00535983
111	K08987	0.023015114	0.132660401	0.025645001	0.027333768	0.010751897	0.017158822	0.007576948	0.008934714
112	K09011	0.026861093	0.127587965	0.000280735	0.000758003	0.000177284	0.000360847	0.001086445	0.001701473
113	K09134	0.012610146	0.152130406	0.01749162	0.014745925	0.008716155	0.009223437	0.006440531	0.007126763
114	K09139	0.024918428	0.130055905	0.000102182	0.000421307	4.08E-05	0.000177657	0.00188744	0.003996517
115	K09155	0.031140121	0.122708014	0.015218587	0.024836177	0.003225832	0.005141408	0.003723141	0.005518831
116	K09384	0.034219402	0.119580759	0.033316175	0.041758425	0.011904876	0.011865062	0.014541051	0.014505042
117	K09459	0.040224154	0.114192957	0.000105744	0.000241557	4.67E-05	0.000105252	0.000453124	0.00086795
118	K09702	0.026442502	0.12810488	0.007149742	0.013057355	0.000564448	0.000812535	0.001613805	0.003160064
119	K09748	0.017809917	0.141011713	0.04339319	0.023785582	0.024634435	0.01900873	0.025482273	0.021294721
120	K09749	0.030592869	0.123294779	0.000696808	0.001204273	0.000327262	0.000606058	0.00310496	0.005827736
121	K09766	0.038206437	0.115911547	0.000592969	0.001110501	0.000144585	0.000297486	0.002733974	0.00560199

				CeD		DC		FDR	
Sr.No	KO_ID	p-values	Effect size	mean rel. freq. (%)	std. dev. (%)	mean rel. freq. (%)	std. dev. (%)	mean rel. freq. (%)	std. dev. (%)
122	K09797	0.031908306	0.121900889	0.000118373	0.000325111	0.000261097	0.000629604	0.001976511	0.004004725
123	K09861	0.032567106	0.12122345	0.037399101	0.024552744	0.022844839	0.018856541	0.01968538	0.015781738
124	K09963	0.033846451	0.119944767	0.04138071	0.05921338	0.013352696	0.021625237	0.010896287	0.011227069
125	K10439	0.038057546	0.116041803	0.033960981	0.030119292	0.017803873	0.015254987	0.017725025	0.013420724
126	K10563	0.049283118	0.107377446	0.042469784	0.025206239	0.026349937	0.019289081	0.026704312	0.019906175
127	K11069	0.015206392	0.146119266	0.040709592	0.026424346	0.022354873	0.014392289	0.023978395	0.018626037
128	K11070	0.010256331	0.158715098	0.039909122	0.02460156	0.021386473	0.013631821	0.023619704	0.018655349
129	K11071	0.015996775	0.144484979	0.038471547	0.023863127	0.021206163	0.013339047	0.023610397	0.018658626
130	K11072	0.006698234	0.172132627	0.039467552	0.024410539	0.019881382	0.013077605	0.023601551	0.018668795
131	K11145	0.042718922	0.112179245	0.032004354	0.030054537	0.013483698	0.016929778	0.018916266	0.018607914
132	K11691	0.024885386	0.130099462	7.74E-05	0.000175083	2.79E-05	9.11E-05	0.001832974	0.003940867
133	K12454	0.043687196	0.111428028	0.000121764	0.00024403	9.04E-05	0.000299587	0.000728899	0.001440439
134	K12555	0.039854689	0.114501351	0.025260826	0.026180239	0.00971718	0.016497771	0.011612875	0.012469581
135	K12556	0.028602329	0.125517755	0.007056073	0.012227702	0.001718904	0.002854261	0.000639848	0.001192638
136	K12999	0.039915631	0.114450294	0.009514745	0.018859219	0.001540887	0.002360639	0.000719932	0.001276392
137	K13012	0.007924336	0.166864615	0.00860426	0.014635483	0.000540046	0.001346611	0.000655353	0.001281055
138	K13075	0.030790539	0.12308168	0.000567279	0.001223444	0.000128306	0.000374696	0.002281746	0.00435504
139	K13677	0.029672435	0.124304838	0.007052636	0.012222697	0.001720914	0.002856318	0.000690109	0.001287778
140	K14230	0.029826249	0.124133967	0.26268206	0.09222215	0.212929063	0.071744719	0.191254601	0.058666829
141	K14231	0.012702851	0.151896018	0.108653184	0.051538349	0.077814518	0.022602861	0.077790474	0.018860257
142	K15524	0.037107052	0.116885075	0.001622436	0.003234418	0.000483473	0.000996923	0.003471886	0.005174199
143	K16012	0.013809329	0.149218902	0.032308857	0.022095327	0.016619002	0.014277979	0.017624935	0.013814687
144	K16013	0.014194976	0.148334151	0.032229945	0.022069162	0.016648274	0.014234918	0.017594521	0.013757881
145	K16149	0.035764839	0.118111977	0	0	3.06E-05	0.000133946	0.001669679	0.003860756
146	K16788	0.025488337	0.129313234	0.007234082	0.012164347	0.001812289	0.00313283	0.000681108	0.001186904
147	K16899	0.036092952	0.117808011	0.032518637	0.030139928	0.013894305	0.017072221	0.017571076	0.017959484
148	K16924	0.007824526	0.167263023	0.016953666	0.021517313	0.005251743	0.006500078	0.003430173	0.003882527
149	K17073	0.026779224	0.127688452	0.014730051	0.023241065	0.004720873	0.005738483	0.002050747	0.003415894
150	K17074	0.025530516	0.129258906	0.014800297	0.023203203	0.004735526	0.005738848	0.002050747	0.003415894
151	K17244	0.007992032	0.166597135	5.90E-05	0.000132365	3.26E-05	0.000106848	0.002993209	0.005640599
152	K17245	0.007992032	0.166597135	5.90E-05	0.000132365	3.26E-05	0.000106848	0.002993209	0.005640599
153	K17246	0.007992032	0.166597135	5.90E-05	0.000132365	3.26E-05	0.000106848	0.002993209	0.005640599
154	K17723	0.049248933	0.107400819	0.002513844	0.003769763	0.003522521	0.004558513	0.007578774	0.008941075
155	K18891	0.046872674	0.109064986	0.017999988	0.023817486	0.005843588	0.007020647	0.007303686	0.012092239
156	K18892	0.046876264	0.109062411	0.017999988	0.023817486	0.005843794	0.007020727	0.007303686	0.012092239
157	K19180	0.041283521	0.113323579	0.003316993	0.003961739	0.005326142	0.00524249	0.010690755	0.013557914
158	K19997	0.03290655	0.120879534	0.000274555	0.000514193	0.000192872	0.000461289	0.001299627	0.00234425
159	K20073	0.024367566	0.130789454	0.006342172	0.010428927	0.001674508	0.002810423	0.000630929	0.00117809
