# Supplementary material for: Comparison of Small Gut and Whole Gut Microbiota of First-Degree Relatives With Adult Celiac Disease Patients and Controls
Source: Front Microbiol. 2019 Feb 8;10:164. doi: 10.3389/fmicb.2019.00164 (PMC6376745; doi:10.3389/fmicb.2019.00164)
Supplement: TABLE S2 — Significantly different KEGG orthologies (KO) between diagnosis groups in duodenal microbiota. Analyzed using the STAMP statistical tool, ANOVA with post hoc Tukey–kramer test was used to identify statistically different KEGG orthologies between diagnosis groups. [file Data_Sheet_2.PDF]

Supplementary Table 2: Significantly different KEGG orthologies (KO) between diagnosis groups in duodenal microbiota. Analyzed using the STAMP statistical tool, ANOVA with post hoc Tukey-kramer test was used to identify statistically different KEGG orthologies between diagnosis groups.

Significantly Different KOs in Duodenal Microbiota

				**CeD**		**DC**		**FDR**	
Sr.No.	KO id	p-values	Effectsize	mean rel.freq.%	std.dev.%	mean rel.freq%	std.dev %	mean rel.freq%	std.dev.%
1	K00298	0.0278571	0.171763217	5.27E-06	1.97E-05	2.40E-06	5.91E-06	6.93E-05	0.0001188
2	K00703	0.0059041	0.236705351	0.027479844	0.008429692	0.020703608	0.00677201	0.032082747	0.0094044
3	K00984	0.0211075	0.183770152	0.003601145	0.005201106	0.000703812	0.00088965	0.000221436	0.0003009
4	K01477	0.0354361	0.161206524	0.001153821	0.001485893	0.004243631	0.006214041	0.000428902	0.0004981
5	K01524	0.0295659	0.169163935	0.090424728	0.035957413	0.067807263	0.020443011	0.062386773	0.020748
6	K01974	0.0318802	0.16586187	0.001568859	0.001614273	0.00074338	0.000811824	0.000402422	0.0003734
7	K01977	0.0480356	0.14766836	0.271039393	0.038596826	0.261309325	0.042184437	0.227200008	0.0514436
8	K01980	0.0211395	0.183705079	0.276130936	0.035607519	0.261798923	0.038078383	0.227479186	0.0524231
9	K01985	0.0281226	0.171349545	0.285093698	0.031440731	0.272116777	0.03600198	0.245701231	0.0386983
10	K02586	0.0415476	0.154152835	2.97E-05	5.17E-05	9.29E-06	2.34E-05	0.000360598	0.0006694
11	K02591	0.0472093	0.148446407	2.97E-05	5.17E-05	9.29E-06	2.34E-05	0.000437219	0.0008392
12	K03319	0.0427215	0.15291152	0.01729831	0.025176648	0.003730974	0.004822938	0.003277759	0.0038172
13	K03324	0.0043349	0.249015936	0.021713815	0.008506414	0.019514647	0.005378547	0.030388455	0.0093174
14	K03396	0.0262174	0.174403502	5.27E-06	1.97E-05	1.31E-06	4.72E-06	6.93E-05	0.0001188
15	K05979	0.0428151	0.152813945	2.67E-05	7.25E-05	0.000865765	0.001422907	0.000241875	0.000358
16	K05982	0.0486737	0.147076219	0.000893644	0.001197346	0.003586851	0.005336333	0.000637973	0.0005457
17	K06919	0.0423579	0.153292491	0.008036543	0.006042921	0.003493117	0.002508342	0.005070458	0.0042604
18	K06987	0.0174441	0.191918422	0.00892897	0.007028698	0.008826381	0.005311542	0.01665118	0.0092326
19	K07166	0.0277249	0.171970501	0.030744366	0.02138678	0.015397406	0.006727091	0.018116925	0.0123514
20	K07485	0.0325454	0.164954843	0.002914532	0.003034837	0.001040842	0.001110245	0.001063511	0.0009906
21	K09018	0.0324051	0.165144612	0.000880142	0.001036592	0.003680285	0.00515918	0.000663889	0.0005609
22	K09020	0.0406663	0.155106813	0.000246684	0.000323115	0.00317457	0.005375836	0.00042346	0.0004738
23	K09021	0.0414718	0.154234107	0.000246684	0.000323115	0.003167215	0.005379966	0.00042346	0.0004738
24	K09023	0.0414718	0.154234107	0.000246684	0.000323115	0.003167215	0.005379966	0.00042346	0.0004738
25	K09157	0.0280148	0.17151713	0.030817442	0.021574892	0.015406366	0.006690639	0.018134505	0.0123342
26	K09805	0.0332546	0.164006907	0.002848893	0.002627367	0.001001532	0.001282525	0.001311435	0.0012052
27	K09818	0.0452688	0.150325499	9.83E-05	0.000263191	0.00015378	0.000518045	0.000817096	0.0012351
28	K10619	0.0128423	0.204839492	0	0	3.25E-06	1.17E-05	2.40E-05	3.52E-05
29	K10620	0.0128423	0.204839492	0	0	3.25E-06	1.17E-05	2.40E-05	3.52E-05
30	K10621	0.0180963	0.190355798	0	0	8.63E-06	2.19E-05	2.70E-05	3.46E-05
31	K10678	0.0497334	0.146108779	0.00084415	0.001050059	0.003416571	0.005185648	0.000559833	0.0004632
32	K11178	0.0486477	0.147100197	0.000794218	0.001254502	0.003699206	0.005563059	0.000683133	0.0009209
33	K12058	0.0145856	0.199494352	1.45E-05	3.98E-05	0	0	0.00011416	0.0001756
34	K12059	0.0145856	0.199494352	1.45E-05	3.98E-05	0	0	0.00011416	0.0001756
35	K12990	0.0266105	0.173756473	0.004017103	0.003741835	0.00166606	0.001512072	0.001587616	0.0011524
36	K13019	0.0035889	0.256443519	6.26E-06	1.43E-05	8.40E-06	1.62E-05	0.000141257	0.0001904
37	K13276	0.0441806	0.151412937	2.32E-05	3.70E-05	0.000140567	0.000210321	2.75E-05	6.45E-05
38	K14414	0.0199012	0.186294254	0.001553925	0.001613717	0.000641056	0.000640302	0.000382694	0.0003344
39	K15515	0.0477467	0.147938923	0	0	6.49E-06	1.25E-05	2.01E-07	6.67E-07
40	K15536	0.0426912	0.152943183	0.002442977	0.002996427	0.000921811	0.001436739	0.000462522	0.0004299

				**CeD**		**DC**		**FDR**	
Sr.No.	KO id	p-values	Effectsize	mean rel.freq.%	std.dev.%	mean rel.freq%	std.dev %	mean rel.freq%	std.dev.%
41	K16303	0.0128423	0.204839492	0	0	3.25E-06	1.17E-05	2.40E-05	3.52E-05
42	K16840	0.0340923	0.162911443	0.001273451	0.001545474	0.004267277	0.006200686	0.000359325	0.0004262
43	K19138	0.0418046	0.153878255	0.00389122	0.003772861	0.001428114	0.001363988	0.00184352	0.001742
44	K19139	0.0418046	0.153878255	0.00389122	0.003772861	0.001428114	0.001363988	0.00184352	0.001742
45	K19140	0.0418046	0.153878255	0.00389122	0.003772861	0.001428114	0.001363988	0.00184352	0.001742
46	K19173	0.0073167	0.228038931	5.27E-06	1.97E-05	3.22E-06	8.05E-06	0.000108053	0.0001589
47	K19174	0.0073167	0.228038931	5.27E-06	1.97E-05	3.22E-06	8.05E-06	0.000108053	0.0001589
48	K19175	0.0039434	0.252748016	5.46E-06	1.97E-05	1.37E-05	1.53E-05	0.000136005	0.0001813
49	K19424	0.046752	0.148882618	1.56E-05	3.23E-05	6.99E-05	7.35E-05	3.33E-05	5.37E-05
50	K20885	0.0464528	0.149170147	0	0	3.68E-07	1.33E-06	8.48E-05	0.0001699
51	K21601	0.0388252	0.157164438	2.53E-06	9.48E-06	5.62E-05	7.49E-05	2.10E-05	5.37E-05
