# Supplementary material for: Comparison of Small Gut and Whole Gut Microbiota of First-Degree Relatives With Adult Celiac Disease Patients and Controls
Source: Front Microbiol. 2019 Feb 8;10:164. doi: 10.3389/fmicb.2019.00164 (PMC6376745; doi:10.3389/fmicb.2019.00164)
Supplement: TABLE S3 — Details of the study subjects including disease status, Marsh values, age, gender, and tTg titre. [file Data_Sheet_3.PDF]

Supplementary Table 3: Details of the study subjects including disease status, Marsh values, age, gender, tTg titre.

Sr. No.	#SampleID	Age	sex	Diagnosis	Ttg	HLA	Marsh	Biopsy	Stool
1	meta_042	14	Male	CeD	296	Positive	3c	YES	YES
2	meta_043	20	Male	CeD	211.78	Positive	3b	YES	YES
3	meta_045	21	Male	CeD	180.12	Positive	3b	YES	YES
4	meta_049	13	Male	CeD	241.77	Positive	3c	YES	YES
5	meta_051	14	Female	CeD	299.9	Positive	3c	NO	YES
6	meta_054	35	Female	FDR	5.57	Positive	0	YES	YES
7	meta_079	55	Male	DC	0.34	Positive	0	YES	YES
8	meta_088	30	Male	DC	1.18	Negative	0	YES	YES
9	meta_089	40	Male	DC	3.2	Positive	0	Yes	NO
10	meta_094	40	Female	FDR	3.98	Positive	0	NO	YES
11	meta_097	20	Male	DC	6.48	Negative	0	YES	YES
12	meta_099	26	Male	DC	6.7	Negative	0	Yes	YES
13	meta_103	20	Male	DC	1	Positive	0	Yes	YES
14	meta_110	35	Male	FDR	4.29	Positive	0	YES	YES
15	meta_114	17	Male	FDR	3.86	Negative	0	YES	YES
16	meta_116	42	Male	FDR	8.87	Positive	0	YES	YES
17	meta_118	22	Female	CeD	214.7	Positive	3c	YES	YES
18	meta_126	38	Female	FDR	9.98	Negative	0	YES	YES
19	meta_131	13	Female	CeD	295	Positive	3c	YES	YES
20	meta_138	47	Female	DC	10.48	Negative	0	YES	YES
21	meta_139	38	Male	DC	8.65	Negative	0	YES	YES
22	meta_141	38	Male	CeD	102	Positive	3c	YES	YES
23	meta_143	12	Male	FDR	0.19	Positive	0	YES	YES
24	meta_145	20	Male	CeD	146.51	Positive	3b	YES	YES
25	meta_146	20	Male	FDR	4.76	Positive	0	YES	YES
26	meta_149	19	Female	CeD	152.1	Positive	3c	YES	NO
27	meta_150	32	Female	CeD	137.7	Positive	3c	YES	NO
28	meta_151	42	Male	DC	1.62	Negative	0	YES	YES
29	meta_152	23	Male	CeD	193.02	Positive	3c	YES	YES
30	meta_157	40	Male	DC	1.18	Negative	1	YES	YES
31	meta_159	21	Female	CeD	128.9	Positive	3c	YES	YES
32	meta_160	28	Male	DC	0.56	Positive	0	NO	YES
33	meta_161	40	Female	FDR	5.29	Positive	0	YES	YES
34	meta_162	20	Male	DC	4.77	Negative	0	YES	YES
35	meta_163	38	Female	FDR	3.27	Positive	0	YES	YES
36	meta_164	17	Female	FDR	2.56	Positive	0	YES	YES
37	meta_165	20	Male	FDR	2.12	Positive	0	YES	YES
38	meta_169	39	Female	FDR	6.38	Positive	0	YES	YES
39	meta_170	27	Male	DC	5.65	Negative	0	YES	YES
40	meta_173	22	Male	DC	2.85	Positive	0	YES	YES
41	meta_174	16	Male	DC	4.88	Negative	0	YES	YES
42	meta_175	27	Male	DC	1.85	Positive	0	NO	YES
43	meta_176	35	Female	CeD	97.4	Positive	3c	YES	YES
44	meta_177	12	Female	CeD	130.2	Positive	3c	YES	YES
45	meta_178	12	Female	CeD	131.3	Positive	3b	YES	YES
46	meta_181	18	Male	CeD	335.7	Positive	3c	YES	YES

47	meta_182	43	Female	FDR	2.3	Positive	0	YES	YES
48	meta_193	30	Female	CeD	288.7	Positive	3b	NO	YES
49	meta_195	37	Female	CeD	106.66	Positive	3b	NO	YES
50	meta_199	40	Female	DC	2.26	Negative	0	NO	YES
51	meta_200	23	Male	DC	2.01	Negative	0	NO	YES
52	meta_204	38	Female	FDR	1.98	Positive	0	NO	YES
53	meta_205	38	Female	CeD	215.5	Positive	3a	NO	YES
54	meta_206	39	Male	DC	2.69	Negative	0	NO	YES
55	meta_209	44	Male	DC	3.82	Negative	0	NO	YES
56	meta_210	26	Male	CeD	208.26	Positive	3a	NO	YES
57	meta_211	20	Male	DC	8.76	Negative	1	NO	YES
58	meta_213	18	Male	DC	7.74	Negative	0	NO	YES
59	meta_214	23	Male	DC	3.65	Negative	0	NO	YES
60	meta_216	43	Male	CeD	220	Positive	3c	NO	YES
61	meta_220	18	Female	CeD	264.67	Positive	3b	NO	YES
62	meta_338	NA	Male	DC	3.67	Negative	0	NO	YES
