# Supplementary material for: Comparison of Small Gut and Whole Gut Microbiota of First-Degree Relatives With Adult Celiac Disease Patients and Controls
Source: Front Microbiol. 2019 Feb 8;10:164. doi: 10.3389/fmicb.2019.00164 (PMC6376745; doi:10.3389/fmicb.2019.00164)
Supplement: TABLE S4 — Number of sequencing reads per sample at each stage of data analysis is given below. [file Data_Sheet_4.PDF]

Supplementary Table 4: Number of sequencing reads per sample at each stage of data analysis is given below.

Sr. No	Sample_ID	No. of reads per sample	No. of reads per sample after filter and trimming	Number of ASVs after taxonomic assignment and discarding chimeric, contaminated, chloroplast and Mitochondrial Seq
1	CB103F	981889	909349	87163
2	CB110F	618248	563576	52119
3	CB114F	791585	737662	80437
4	CB116F	783377	728102	69055
5	CB118F	826384	771662	81295
6	CB126F	578299	543313	59923
7	CB131F	642474	597873	59510
8	CB138F	503394	468565	51279
9	CB139F	743426	692543	66276
10	CB141F	463343	433409	63376
11	CB143F	574035	542301	120710
12	CB145F	620760	577472	136915
13	CB146F	522187	487644	60587
14	CB149F	353492	329358	37802
15	CB150F	540162	503535	120549
16	CB151F	534622	495907	117627
17	CB152F	593507	550724	90758
18	CB157F	642837	601295	101384
19	CB159F	655638	609048	60275
20	CB161F	551514	514154	130927
21	CB162F	506295	466104	29185
22	CB163F	868475	809932	87508
23	CB164F	1289408	1196256	123224
24	CB165F	867600	804157	93346
25	CB169F	459735	427009	32404
26	CB170F	718946	668898	118910
27	CB173F	1009744	937601	101756
28	CB174F	422921	390023	48112
29	CB176F	478864	445244	36847
30	CB177F	1286072	1207169	124841
31	CB178F	594974	553664	45559
32	CB181F	836918	780423	85818
33	CB182F	433899	403984	34685
34	CB42F	948320	887928	55344
35	CB43F	996714	923214	81745
36	CB45F	1055134	924204	77005
37	CB49F	638665	604464	141745
38	CB54F	561606	530944	69879
39	CB79F	1107413	1030854	101882
40	CB88F	1244546	1174073	131605
41	CB89F	897179	834257	85088
42	CB97F	923903	868573	107534
43	CB99F	1020606	945488	98109
44	CS103F	709724	640385	34644

Supplementary table 2: Number of reads per sample at each stage of analsyis

45	CS110F	929502	651198	38547
46	CS114F	1048361	980860	97358
47	CS116F	652271	614505	37446
48	CS118F	641477	604070	30333
49	CS126F	719687	657522	51572
50	CS131F	636390	606530	34155
51	CS138F	799235	715403	54155
52	CS139F	691892	611219	48182
53	CS141F	612423	529601	44999
54	CS143F	1020404	898710	34339
55	CS145F	680418	617556	37760
56	CS146F	662303	598114	50481
57	CS151F	567019	509667	44637
58	CS152F	721968	684782	31276
59	CS157F	721952	681032	43863
60	CS159F	755522	717684	46064
61	CS160F	903147	853768	67954
62	CS161F	791129	747763	57442
63	CS162F	695997	663129	47743
64	CS163F	739480	692992	50917
65	CS164F	673493	633567	42296
66	CS165F	681530	642335	42312
67	CS169F	660564	624112	40774
68	CS170F	668906	628051	42001
69	CS173F	596470	565084	36388
70	CS174F	784955	738546	61844
71	CS175F	730964	689130	97461
72	CS176F	638197	603552	68505
73	CS177F	844257	800728	61850
74	CS178F	1161760	1061343	100310
75	CS181F	847217	799757	51768
76	CS182F	1030678	965316	102711
77	CS193F	615493	582474	38484
78	CS195F	683391	646156	47213
79	CS199F	739065	692614	52322
80	CS200F	960944	895596	64911
81	CS204F	880142	827877	42923
82	CS205F	943109	887415	54644
83	CS206F	752798	707054	94550
84	CS209F	636880	602139	61872
85	CS210F	797975	749932	61342
86	CS211F	718001	680609	37062
87	CS213F	710778	674496	68761
88	CS214F	929285	868380	72140
89	CS216F	779604	736063	69795
90	CS220F	606844	571743	36178
91	CS338F	591938	560999	44202
92	CS42F	590287	497180	38385

Supplementary table 2: Number of reads per sample at each stage of analsyis

93	CS43F	364517	325350	6278
94	CS45F	731040	676253	40839
95	CS49F	1076791	1013135	65625
96	CS51F	784407	741301	30201
97	CS54F	873362	813642	63343
98	CS79F	582320	504406	15196
99	CS88F	950774	886144	50503
100	CS94F	639036	604599	20755
101	CS97F	750541	678222	63030
102	CS99F	660328	579202	34405
	Total	76058052	70502947	6567144
	Average	745667	691205	64383
