# Supplementary material for: Comparison of Small Gut and Whole Gut Microbiota of First-Degree Relatives With Adult Celiac Disease Patients and Controls
Source: Front Microbiol. 2019 Feb 8;10:164. doi: 10.3389/fmicb.2019.00164 (PMC6376745; doi:10.3389/fmicb.2019.00164)
Supplement: INFORMATION — Differential Abundance of Amplicon Sequence Variants of Helicobacter. Multiple sequence alignment was performed by CLUSTAL 2.0.11. [file Data_Sheet_5.PDF]

Supplementary material

Comparison of Small Gut and Whole Gut Microbiota of First-Degree Relatives with Adult Celiac Disease Patients and Controls

Rahul Bodkhe[1], Sudarshan A. Shetty[1,5], Dhiraj P. Dhotre[1], Anil K. Verma[2,6], Khushbo Bhatia[2], Asha Mishra[2], Gurvinder Kaur[3], Pranav Pande[1], Dhinoth K. Bangarusamy[4], Beena P. Santosh[4], Rajadurai C. Perumal[4], Vineet Ahuja[2], Yogesh S. Shouche[1*], Govind K. Makharia[2*]

[1] National Centre for Microbial Resource, National Centre for Cell Science, Pune-411007, India.
[2] Department of Gastroenterology and Human Nutrition, All India Institute of Medical Sciences, New Delhi, India.
3 Department of transplant Immunology & Immunogenetics, All India Institute of Medical Sciences, New Delhi, India
[4] AgriGenome Labs Pvt. Ltd. Kerala, India.
[5] Current address: Laboratory of Microbiology, Wageningen University and Research, Building 124, Stippeneng 4, 6708 WE Wageningen, The Netherlands.
[6] Current address: Celiac Disease Research Laboratory, Department of Pediatrics, Università Politecnica delle Marche, Ancona, Italy.

*Co-corresponding authors: Yogesh S. Shouche (yogesh@nccs.res.in) and Govind K. Makharia (govindmakharia@gmail.com)

Supplementary Information Table 1: Differential Abundance of Amplicon Sequence Variant of *H. pylori*

ASV ID	baseMean	log2FoldChange	pvalue	padj	Species	Comparison	Diff. Abundant in
**ASV1811**	39.59522	-25.1038	1.31E-17	1.06E-15	H. pylori	CeD vs DC	CeD Supplementary Figure 1A
**ASV1811**	40.24889	-24.9026	4.29E-17	2.16E-15	H. pylori	CeD vs FDR	CeD Supplementary Figure 1B
**ASV2016**	32.63937	24.44444	1.05E-16	4.97E-15	H. pylori	FDR vs DC	FDR Supplementary Figure 1C
**ASV4095**	16.22696	23.4685	1.63E-15	2.53E-14	H. pylori	FDR vs DC	FDR Supplementary Figure 1C

Supplementary Information Figure 1: Differential Abundance of *H. pylori* Amplicon Sequence Variants highlighted in duodenal microbiota comparison between diagnosis groups

A

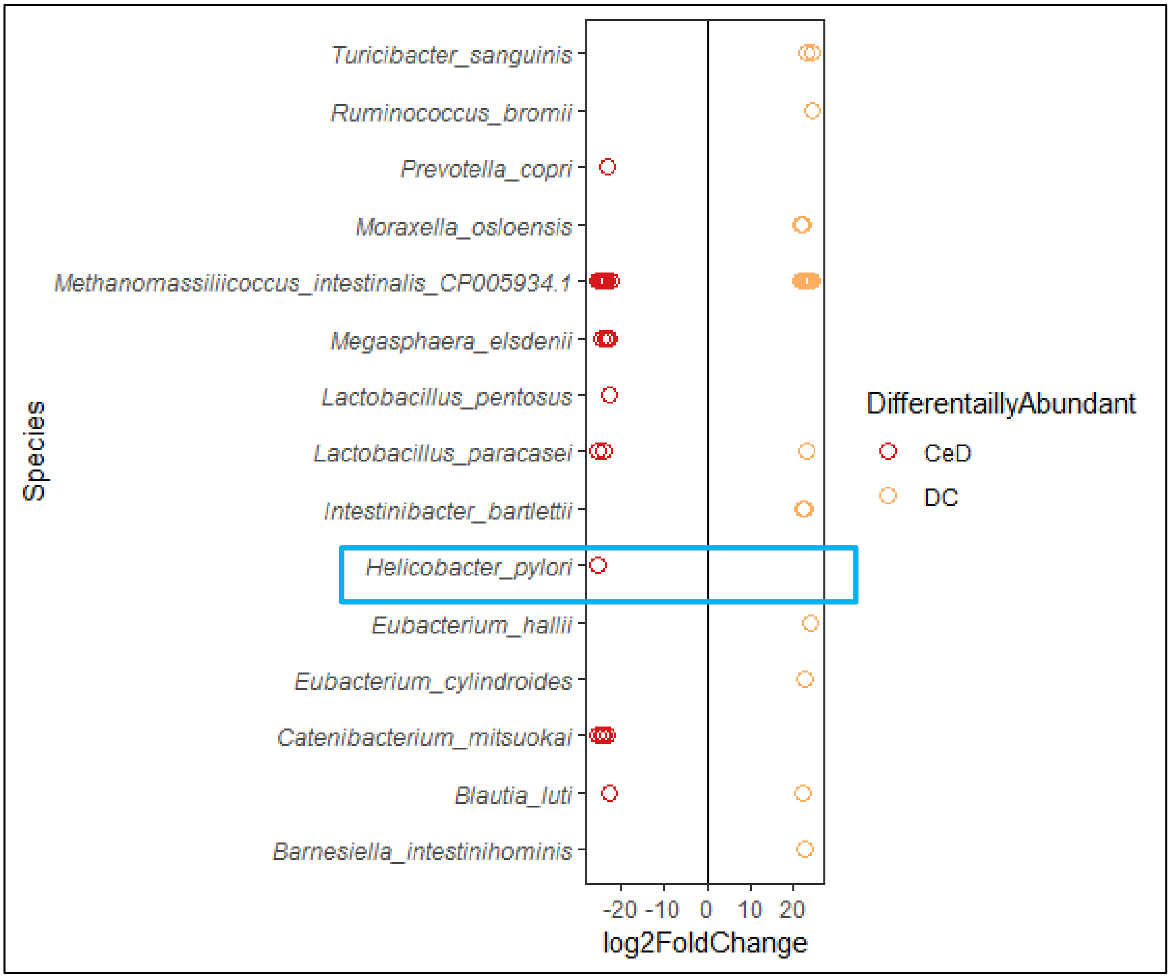

Supplementary Information Figure 1: Differential Abundance of *H. pylori* Amplicon Sequence Variants highlighted in duodenal microbiota comparison between diagnosis groups

B

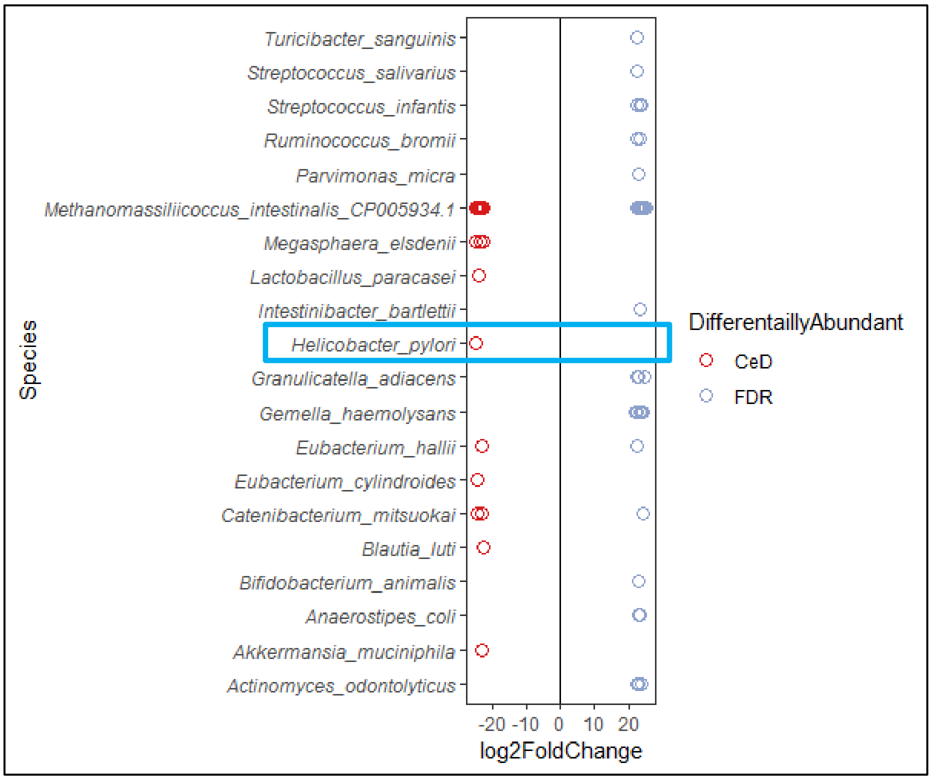

C

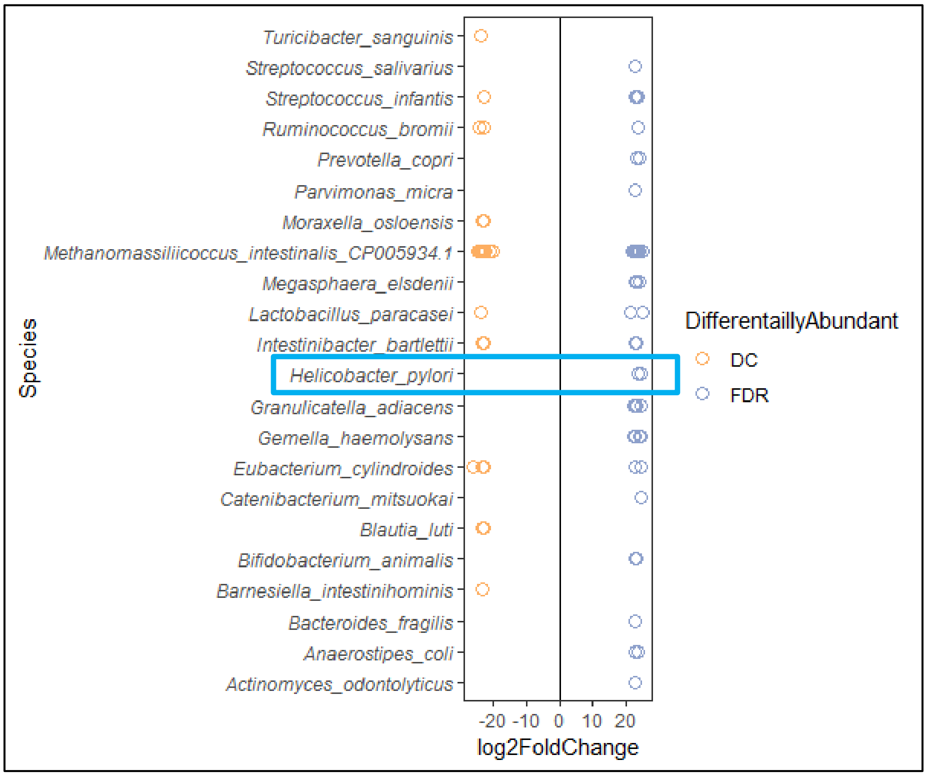

Supplementary information

Multiple alignment of the V4 region of 16S rRNA gene for publicly *H. pylori* 16S rRNA gene sequences

Page 1 of 5

```
                                                  ************************************* ******** ************* **
NZ_CP024016_H_pylori_7.13_D1b_copy2 -----------------TGCCAGCAGCCGCGGTAATACGGAGGGTGCAAGCGTTACTCGGAATCACTGGGCGTAAAGAGC    80
NZ_CP028325_H_pylori_FDAARGOS_298   -----------------TGCCAGCAGCCGCGGTAATACGGAGGGTGCAAGCGTTACTCGGAATCACTGGGCGTAAAGAGC    80
NZ_CP022409_H_pylori_G272           -----------------TGCCAGCAGCCGCGGTAATACGGAGGGTGCAAGCGTTACTCGGAATCACTGGGCGTAAAGAGC    80
NZ_LT635459_H_pylori_HE132/09S      -----------------TGCCAGCAGCCGCGGTAATACGGAGGGTGCAAGCGTTACTCGGAATCACTGGGCGTAAAGAGC    80
NZ_LT635471_H_pylori_HE141/09       -----------------TGCCAGCAGCCGCGGTAATACGGAGGGTGCAAGCGTTACTCGGAATCACTGGGCGTAAAGAGC    80
NZ_LT635474_H_pylori_HE171/09       -----------------TGCCAGCAGCCGCGGTAATACGGAGGGTGCAAGCGTTACTCGGAATCACTGGGCGTAAAGAGC    80
NC_017372_H_pylori_India7_          -----------------TGCCAGCAGCCGCGGTAATACGGAGGGTGCAAGCGTTACTCGGAATCACTGGGCGTAAAGAGC    80
NZ_CP006822_H_pylori_oki128_        -----------------TGCCAGCAGCCGCGGTAATACGGAGGGTGCAAGCGTTACTCGGAATCACTGGGCGTAAAGAGC    80
NC_021215.3_H_pylori_UM032_copy2    -----------------TGCCAGCAGCCGCGGTAATACGGAGGGTGCAAGCGTTACTCGGAATCACTGGGCGTAAAGAGC    80
NC_021216.3_H_pylori_UM299          -----------------TGCCAGCAGCCGCGGTAATACGGAGGGTGCAAGCGTTACTCGGAATCACTGGGCGTAAAGAGC    80
CP024017_H_pylori_7.13_D1c          -----------------TGCCAGCAGCCGCGGTAATACGGAGGGTGCAAGCGTTACTCGGAATCACTGGGCGTAAAGAGC    80
CP024017_H_pylori_7.13_D1c_copy2    -----------------TGCCAGCAGCCGCGGTAATACGGAGGGTGCAAGCGTTACTCGGAATCACTGGGCGTAAAGAGC    80
NZ_CP024021_H_pylori_7.13_D3a       -----------------TGCCAGCAGCCGCGGTAATACGGAGGGTGCAAGCGTTACTCGGAATCACTGGGCGTAAAGAGC    80
NZ_CP024021_H_pylori_7.13_D3a_copy2 -----------------TGCCAGCAGCCGCGGTAATACGGAGGGTGCAAGCGTTACTCGGAATCACTGGGCGTAAAGAGC    80
NZ_CP024022_H_pylori_7.13_D3b       -----------------TGCCAGCAGCCGCGGTAATACGGAGGGTGCAAGCGTTACTCGGAATCACTGGGCGTAAAGAGC    80
NZ_CP024022_H_pylori_7.13_D3b_copy2 -----------------TGCCAGCAGCCGCGGTAATACGGAGGGTGCAAGCGTTACTCGGAATCACTGGGCGTAAAGAGC    80
NZ_CP024023_H_pylori_7.13_D3c       -----------------TGCCAGCAGCCGCGGTAATACGGAGGGTGCAAGCGTTACTCGGAATCACTGGGCGTAAAGAGC    80
NZ_CP024023_H_pylori_7.13_D3c_copy2 -----------------TGCCAGCAGCCGCGGTAATACGGAGGGTGCAAGCGTTACTCGGAATCACTGGGCGTAAAGAGC    80
NZ_CP024071_H_pylori_7.13_R1a       -----------------TGCCAGCAGCCGCGGTAATACGGAGGGTGCAAGCGTTACTCGGAATCACTGGGCGTAAAGAGC    80
NZ_CP024071_H_pylori_7.13_R1a_copy2 -----------------TGCCAGCAGCCGCGGTAATACGGAGGGTGCAAGCGTTACTCGGAATCACTGGGCGTAAAGAGC    80
NZ_CP024072_H_pylori_7.13_R1b       -----------------TGCCAGCAGCCGCGGTAATACGGAGGGTGCAAGCGTTACTCGGAATCACTGGGCGTAAAGAGC    80
NZ_CP024072_H_pylori_7.13_R1b_copy2 -----------------TGCCAGCAGCCGCGGTAATACGGAGGGTGCAAGCGTTACTCGGAATCACTGGGCGTAAAGAGC    80
NZ_CP024073_H_pylori_7.13_R1c       -----------------TGCCAGCAGCCGCGGTAATACGGAGGGTGCAAGCGTTACTCGGAATCACTGGGCGTAAAGAGC    80
NZ_CP024073_H_pylori_7.13_R1c_copy2 -----------------TGCCAGCAGCCGCGGTAATACGGAGGGTGCAAGCGTTACTCGGAATCACTGGGCGTAAAGAGC    80
NZ_CP024074_H_pylori_7.13_R2a       -----------------TGCCAGCAGCCGCGGTAATACGGAGGGTGCAAGCGTTACTCGGAATCACTGGGCGTAAAGAGC    80
NZ_CP024074_H_pylori_7.13_R2a_copy2 -----------------TGCCAGCAGCCGCGGTAATACGGAGGGTGCAAGCGTTACTCGGAATCACTGGGCGTAAAGAGC    80
NZ_CP024075_H_pylori_7.13_R2b       -----------------TGCCAGCAGCCGCGGTAATACGGAGGGTGCAAGCGTTACTCGGAATCACTGGGCGTAAAGAGC    80
NZ_CP024075_H_pylori_7.13_R2b_copy2 -----------------TGCCAGCAGCCGCGGTAATACGGAGGGTGCAAGCGTTACTCGGAATCACTGGGCGTAAAGAGC    80
NZ_CP024076_H_pylori_7.13_R2c       -----------------TGCCAGCAGCCGCGGTAATACGGAGGGTGCAAGCGTTACTCGGAATCACTGGGCGTAAAGAGC    80
NZ_CP024076_H_pylori_7.13_R2c_copy2 -----------------TGCCAGCAGCCGCGGTAATACGGAGGGTGCAAGCGTTACTCGGAATCACTGGGCGTAAAGAGC    80
NZ_CP024077_H_pylori_7.13_R3a       -----------------TGCCAGCAGCCGCGGTAATACGGAGGGTGCAAGCGTTACTCGGAATCACTGGGCGTAAAGAGC    80
NZ_CP024077_H_pylori_7.13_R3a_copy2 -----------------TGCCAGCAGCCGCGGTAATACGGAGGGTGCAAGCGTTACTCGGAATCACTGGGCGTAAAGAGC    80
NZ_CP024078_H_pylori_7.13_R3b       -----------------TGCCAGCAGCCGCGGTAATACGGAGGGTGCAAGCGTTACTCGGAATCACTGGGCGTAAAGAGC    80
NZ_CP024078_H_pylori_7.13_R3b_copy2 -----------------TGCCAGCAGCCGCGGTAATACGGAGGGTGCAAGCGTTACTCGGAATCACTGGGCGTAAAGAGC    80
NZ_CP024079_H_pylori_7.13_R3c       -----------------TGCCAGCAGCCGCGGTAATACGGAGGGTGCAAGCGTTACTCGGAATCACTGGGCGTAAAGAGC    80
NZ_CP024079_H_pylori_7.13_R3c_copy2 -----------------TGCCAGCAGCCGCGGTAATACGGAGGGTGCAAGCGTTACTCGGAATCACTGGGCGTAAAGAGC    80
NC_019563_H_pylori_Aklavik86_       -----------------TGCCAGCAGCCGCGGTAATACGGAGGGTGCAAGCGTTACTCGGAATCACTGGGCGTAAAGAGC    80
NZ_CP011485_H_pylori_ausabrJ05_     -----------------TGCCAGCAGCCGCGGTAATACGGAGGGTGCAAGCGTTACTCGGAATCACTGGGCGTAAAGAGC    80
NC_014256_H_pylori_B8               -----------------TGCCAGCAGCCGCGGTAATACGGAGGGTGCAAGCGTTACTCGGAATCACTGGGCGTAAAGAGC    80
NC_014256_H_pylori_B8_copy2         -----------------TGCCAGCAGCCGCGGTAATACGGAGGGTGCAAGCGTTACTCGGAATCACTGGGCGTAAAGAGC    80
NZ_LT837687_H_pylori_BCM-300        -----------------TGCCAGCAGCCGCGGTAATACGGAGGGTGCAAGCGTTACTCGGAATCACTGGGCGTAAAGAGC    80
NC_022886_H_pylori_BM012A_          -----------------TGCCAGCAGCCGCGGTAATACGGAGGGTGCAAGCGTTACTCGGAATCACTGGGCGTAAAGAGC    80
NZ_CP007605_H_pylori_BM012B_        -----------------TGCCAGCAGCCGCGGTAATACGGAGGGTGCAAGCGTTACTCGGAATCACTGGGCGTAAAGAGC    80
NC_022911_H_pylori_BM012S_          -----------------TGCCAGCAGCCGCGGTAATACGGAGGGTGCAAGCGTTACTCGGAATCACTGGGCGTAAAGAGC    80
NZ_CP011483_H_pylori_DU15_          -----------------TGCCAGCAGCCGCGGTAATACGGAGGGTGCAAGCGTTACTCGGAATCACTGGGCGTAAAGAGC    80
NZ_CP022409_H_pylori_G272_copy2     -----------------TGCCAGCAGCCGCGGTAATACGGAGGGTGCAAGCGTTACTCGGAATCACTGGGCGTAAAGAGC    80
NZ_LT635456_H_pylori_HE101/09       -----------------TGCCAGCAGCCGCGGTAATACGGAGGGTGCAAGCGTTACTCGGAATCACTGGGCGTAAAGAGC    80
NZ_LT635476_H_pylori_HE134/09       -----------------TGCCAGCAGCCGCGGTAATACGGAGGGTGCAAGCGTTACTCGGAATCACTGGGCGTAAAGAGC    80
NZ_LT635473_H_pylori_HE136/09       -----------------TGCCAGCAGCCGCGGTAATACGGAGGGTGCAAGCGTTACTCGGAATCACTGGGCGTAAAGAGC    80
NZ_LT635478_H_pylori_HE142/09       -----------------TGCCAGCAGCCGCGGTAATACGGAGGGTGCAAGCGTTACTCGGAATCACTGGGCGTAAAGAGC    80
NZ_LT635458_H_pylori_HE143/09       -----------------TGCCAGCAGCCGCGGTAATACGGAGGGTGCAAGCGTTACTCGGAATCACTGGGCGTAAAGAGC    80
NZ_LT635477_H_pylori_HE147/09       -----------------TGCCAGCAGCCGCGGTAATACGGAGGGTGCAAGCGTTACTCGGAATCACTGGGCGTAAAGAGC    80
NZ_LT635472_H_pylori_HE170/09       -----------------TGCCAGCAGCCGCGGTAATACGGAGGGTGCAAGCGTTACTCGGAATCACTGGGCGTAAAGAGC    80
NZ_LT635460_H_pylori_HE178/09       -----------------TGCCAGCAGCCGCGGTAATACGGAGGGTGCAAGCGTTACTCGGAATCACTGGGCGTAAAGAGC    80
NZ_LT838273_H_pylori_HE93/10_v1     -----------------TGCCAGCAGCCGCGGTAATACGGAGGGTGCAAGCGTTACTCGGAATCACTGGGCGTAAAGAGC    80
NZ_AP014710_H_pylori_DNA            -----------------TGCCAGCAGCCGCGGTAATACGGAGGGTGCAAGCGTTACTCGGAATCACTGGGCGTAAAGAGC    80
NZ_AP014710_H_pylori_DNA_copy2      -----------------TGCCAGCAGCCGCGGTAATACGGAGGGTGCAAGCGTTACTCGGAATCACTGGGCGTAAAGAGC    80
NZ_AP014712_H_pylori_DNA            -----------------TGCCAGCAGCCGCGGTAATACGGAGGGTGCAAGCGTTACTCGGAATCACTGGGCGTAAAGAGC    80
NZ_AP014712_H_pylori_DNA_copy2      -----------------TGCCAGCAGCCGCGGTAATACGGAGGGTGCAAGCGTTACTCGGAATCACTGGGCGTAAAGAGC    80
NC_017742_H_pylori_PeCan18_         -----------------TGCCAGCAGCCGCGGTAATACGGAGGGTGCAAGCGTTACTCGGAATCACTGGGCGTAAAGAGC    80
NC_021215.3_H_pylori_UM032          -----------------TGCCAGCAGCCGCGGTAATACGGAGGGTGCAAGCGTTACTCGGAATCACTGGGCGTAAAGAGC    80
NC_021882.2_H_pylori_UM298          -----------------TGCCAGCAGCCGCGGTAATACGGAGGGTGCAAGCGTTACTCGGAATCACTGGGCGTAAAGAGC    80
NC_021882.2_H_pylori_UM298_copy2    -----------------TGCCAGCAGCCGCGGTAATACGGAGGGTGCAAGCGTTACTCGGAATCACTGGGCGTAAAGAGC    80
NC_021216.3_H_pylori_UM299_copy2    -----------------TGCCAGCAGCCGCGGTAATACGGAGGGTGCAAGCGTTACTCGGAATCACTGGGCGTAAAGAGC    80
NC_017355_H_pylori_v225d_           -----------------TGCCAGCAGCCGCGGTAATACGGAGGGTGCAAGCGTTACTCGGAATCACTGGGCGTAAAGAGC    80
NC_017365_H_pylori_F30_DNA_         -----------------TGCCAGCAGCCGCGGTAATACGGAGGGTGCAAGCGTTACTCGGAATCACTGGGCGTAAAGAGC    80
NZ_CP024016_H_pylori_7.13_D1b       -----------------TGCCAGCAGCCGCGGTAATACGGAGGGTGCAAGCGTTACTCGGAATCACTGGGCGTAAAGAGC    80
CP024015_H_pylori_7.13_D1a_copy2    -----------------TGCCAGCAGCCGCGGTAATACGGAGGGTGCAAGCGTTACTCGGAATCACTGGGCGTAAAGAGC    80
CP024015_H_pylori_7.13_D1a          -----------------TGCCAGCAGCCGCGGTAATACGGAGGGTGCAAGCGTTACTCGGAATCACTGGGCGTAAAGAGC    80
NR_024570_E_coli_U_5/41             AGCACCGGCTAACTCCGTGCCAGCAGCCGCGGTAATACGGAGGGTGCAAGCGTTAATCGGAATTACTGGGCGTAAAGCGC    80
                                    0.......500.......510.......520.......530.......540.......550.......560.......57
```

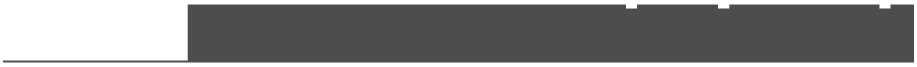

CLUSTAL 2.0.11 MULTIPLE SEQUENCE ALIGNMENT


```
                                      ** ****** * * ****** **********   **** ****   ********* *** ***   * ** ****
NZ_CP024016_H_pylori_7.13_D1b_copy2 GCGTAGGCGGGATAGTCAGTCAGGTGTGAAATCCTATGGCTTAACCATAGAACTGCATTTGAAACTACTATTCTAGAGTG  160
NZ_CP028325_H_pylori_FDAARGOS_298   GCGTAGGCGGGATAGTCAGTCAGGTGTGAAATCCTATGGCTTAACCATAGAACTGCATTTGAAACTACTATTCTAGAGTG  160
NZ_CP022409_H_pylori_G272           GCGTAGGCGGGATAGTCAGTCAGGTGTGAAATCCTATGGCTTAACCATAGAACTGCATTTGAAACTACTATTCTAGAGTG  160
NZ_LT635459_H_pylori_HE132/09S      GCGTAGGCGGGATAGTCAGTCAGGTGTGAAATCCTATGGCTTAACCATAGAACTGCATTTGAAACTACTATTCTAGAGTG  160
NZ_LT635471_H_pylori_HE141/09       GCGTAGGCGGGATAGTCAGTCAGGTGTGAAATCCTATGGCTTAACCATAGAACTGCATTTGAAACTACTATTCTAGAGTG  160
NZ_LT635474_H_pylori_HE171/09       GCGTAGGCGGGATAGTCAGTCAGGTGTGAAATCCTATGGCTTAACCATAGAACTGCATTTGAAACTACTATTCTAGAGTG  160
NC_017372_H_pylori_India7_          GCGTAGGCGGGATAGTCAGTCAGGTGTGAAATCCTATGGCTTAACCATAGAACTGCATTTGAAACTACTATTCTAGAGTG  160
NZ_CP006822_H_pylori_oki128_        GCGTAGGCGGGATAGTCAGTCAGGTGTGAAATCCTATGGCTTAACCATAGAACTGCATTTGAAACTACTATTCTAGAGTG  160
NC_021215.3_H_pylori_UM032_copy2    GCGTAGGCGGGATAGTCAGTCAGGTGTGAAATCCTATGGCTTAACCATAGAACTGCATTTGAAACTACTATTCTAGAGTG  160
NC_021216.3_H_pylori_UM299          GCGTAGGCGGGATAGTCAGTCAGGTGTGAAATCCTATGGCTTAACCATAGAACTGCATTTGAAACTACTATTCTAGAGTG  160
CP024017_H_pylori_7.13_D1c          GCGTAGGCGGGATAGTCAGTCAGGTGTGAAATCCTATGGCTTAACCATAGAACTGCATTTGAAACTACTATTCTAGAGTG  160
CP024017_H_pylori_7.13_D1c_copy2    GCGTAGGCGGGATAGTCAGTCAGGTGTGAAATCCTATGGCTTAACCATAGAACTGCATTTGAAACTACTATTCTAGAGTG  160
NZ_CP024021_H_pylori_7.13_D3a       GCGTAGGCGGGATAGTCAGTCAGGTGTGAAATCCTATGGCTTAACCATAGAACTGCATTTGAAACTACTATTCTAGAGTG  160
NZ_CP024021_H_pylori_7.13_D3a_copy2 GCGTAGGCGGGATAGTCAGTCAGGTGTGAAATCCTATGGCTTAACCATAGAACTGCATTTGAAACTACTATTCTAGAGTG  160
NZ_CP024022_H_pylori_7.13_D3b       GCGTAGGCGGGATAGTCAGTCAGGTGTGAAATCCTATGGCTTAACCATAGAACTGCATTTGAAACTACTATTCTAGAGTG  160
NZ_CP024022_H_pylori_7.13_D3b_copy2 GCGTAGGCGGGATAGTCAGTCAGGTGTGAAATCCTATGGCTTAACCATAGAACTGCATTTGAAACTACTATTCTAGAGTG  160
NZ_CP024023_H_pylori_7.13_D3c       GCGTAGGCGGGATAGTCAGTCAGGTGTGAAATCCTATGGCTTAACCATAGAACTGCATTTGAAACTACTATTCTAGAGTG  160
NZ_CP024023_H_pylori_7.13_D3c_copy2 GCGTAGGCGGGATAGTCAGTCAGGTGTGAAATCCTATGGCTTAACCATAGAACTGCATTTGAAACTACTATTCTAGAGTG  160
NZ_CP024071_H_pylori_7.13_R1a       GCGTAGGCGGGATAGTCAGTCAGGTGTGAAATCCTATGGCTTAACCATAGAACTGCATTTGAAACTACTATTCTAGAGTG  160
NZ_CP024071_H_pylori_7.13_R1a_copy2 GCGTAGGCGGGATAGTCAGTCAGGTGTGAAATCCTATGGCTTAACCATAGAACTGCATTTGAAACTACTATTCTAGAGTG  160
NZ_CP024072_H_pylori_7.13_R1b       GCGTAGGCGGGATAGTCAGTCAGGTGTGAAATCCTATGGCTTAACCATAGAACTGCATTTGAAACTACTATTCTAGAGTG  160
NZ_CP024072_H_pylori_7.13_R1b_copy2 GCGTAGGCGGGATAGTCAGTCAGGTGTGAAATCCTATGGCTTAACCATAGAACTGCATTTGAAACTACTATTCTAGAGTG  160
NZ_CP024073_H_pylori_7.13_R1c       GCGTAGGCGGGATAGTCAGTCAGGTGTGAAATCCTATGGCTTAACCATAGAACTGCATTTGAAACTACTATTCTAGAGTG  160
NZ_CP024073_H_pylori_7.13_R1c_copy2 GCGTAGGCGGGATAGTCAGTCAGGTGTGAAATCCTATGGCTTAACCATAGAACTGCATTTGAAACTACTATTCTAGAGTG  160
NZ_CP024074_H_pylori_7.13_R2a       GCGTAGGCGGGATAGTCAGTCAGGTGTGAAATCCTATGGCTTAACCATAGAACTGCATTTGAAACTACTATTCTAGAGTG  160
NZ_CP024074_H_pylori_7.13_R2a_copy2 GCGTAGGCGGGATAGTCAGTCAGGTGTGAAATCCTATGGCTTAACCATAGAACTGCATTTGAAACTACTATTCTAGAGTG  160
NZ_CP024075_H_pylori_7.13_R2b       GCGTAGGCGGGATAGTCAGTCAGGTGTGAAATCCTATGGCTTAACCATAGAACTGCATTTGAAACTACTATTCTAGAGTG  160
NZ_CP024075_H_pylori_7.13_R2b_copy2 GCGTAGGCGGGATAGTCAGTCAGGTGTGAAATCCTATGGCTTAACCATAGAACTGCATTTGAAACTACTATTCTAGAGTG  160
NZ_CP024076_H_pylori_7.13_R2c       GCGTAGGCGGGATAGTCAGTCAGGTGTGAAATCCTATGGCTTAACCATAGAACTGCATTTGAAACTACTATTCTAGAGTG  160
NZ_CP024076_H_pylori_7.13_R2c_copy2 GCGTAGGCGGGATAGTCAGTCAGGTGTGAAATCCTATGGCTTAACCATAGAACTGCATTTGAAACTACTATTCTAGAGTG  160
NZ_CP024077_H_pylori_7.13_R3a       GCGTAGGCGGGATAGTCAGTCAGGTGTGAAATCCTATGGCTTAACCATAGAACTGCATTTGAAACTACTATTCTAGAGTG  160
NZ_CP024077_H_pylori_7.13_R3a_copy2 GCGTAGGCGGGATAGTCAGTCAGGTGTGAAATCCTATGGCTTAACCATAGAACTGCATTTGAAACTACTATTCTAGAGTG  160
NZ_CP024078_H_pylori_7.13_R3b       GCGTAGGCGGGATAGTCAGTCAGGTGTGAAATCCTATGGCTTAACCATAGAACTGCATTTGAAACTACTATTCTAGAGTG  160
NZ_CP024078_H_pylori_7.13_R3b_copy2 GCGTAGGCGGGATAGTCAGTCAGGTGTGAAATCCTATGGCTTAACCATAGAACTGCATTTGAAACTACTATTCTAGAGTG  160
NZ_CP024079_H_pylori_7.13_R3c       GCGTAGGCGGGATAGTCAGTCAGGTGTGAAATCCTATGGCTTAACCATAGAACTGCATTTGAAACTACTATTCTAGAGTG  160
NZ_CP024079_H_pylori_7.13_R3c_copy2 GCGTAGGCGGGATAGTCAGTCAGGTGTGAAATCCTATGGCTTAACCATAGAACTGCATTTGAAACTACTATTCTAGAGTG  160
NC_019563_H_pylori_Aklavik86_       GCGTAGGCGGGATAGTCAGTCAGGTGTGAAATCCTATGGCTTAACCATAGAACTGCATTTGAAACTACTATTCTAGAGTG  160
NZ_CP011485_H_pylori_ausabrJ05_     GCGTAGGCGGGATAGTCAGTCAGGTGTGAAATCCTATGGCTTAACCATAGAACTGCATTTGAAACTACTATTCTAGAGTG  160
NC_014256_H_pylori_B8               GCGTAGGCGGGATAGTCAGTCAGGTGTGAAATCCTATGGCTTAACCATAGAACTGCATTTGAAACTACTATTCTAGAGTG  160
NC_014256_H_pylori_B8_copy2         GCGTAGGCGGGATAGTCAGTCAGGTGTGAAATCCTATGGCTTAACCATAGAACTGCATTTGAAACTACTATTCTAGAGTG  160
NZ_LT837687_H_pylori_BCM-300        GCGTAGGCGGGATAGTCAGTCAGGTGTGAAATCCTATGGCTTAACCATAGAACTGCATTTGAAACTACTATTCTAGAGTG  160
NC_022886_H_pylori_BM012A_          GCGTAGGCGGGATAGTCAGTCAGGTGTGAAATCCTATGGCTTAACCATAGAACTGCATTTGAAACTACTATTCTAGAGTG  160
NZ_CP007605_H_pylori_BM012B_        GCGTAGGCGGGATAGTCAGTCAGGTGTGAAATCCTATGGCTTAACCATAGAACTGCATTTGAAACTACTATTCTAGAGTG  160
NC_022911_H_pylori_BM012S_          GCGTAGGCGGGATAGTCAGTCAGGTGTGAAATCCTATGGCTTAACCATAGAACTGCATTTGAAACTACTATTCTAGAGTG  160
NZ_CP011483_H_pylori_DU15_          GCGTAGGCGGGATAGTCAGTCAGGTGTGAAATCCTATGGCTTAACCATAGAACTGCATTTGAAACTACTATTCTAGAGTG  160
NZ_CP022409_H_pylori_G272_copy2     GCGTAGGCGGGATAGTCAGTCAGGTGTGAAATCCTATGGCTTAACCATAGAACTGCATTTGAAACTACTATTCTAGAGTG  160
NZ_LT635456_H_pylori_HE101/09       GCGTAGGCGGGATAGTCAGTCAGGTGTGAAATCCTATGGCTTAACCATAGAACTGCATTTGAAACTACTATTCTAGAGTG  160
NZ_LT635476_H_pylori_HE134/09       GCGTAGGCGGGATAGTCAGTCAGGTGTGAAATCCTATGGCTTAACCATAGAACTGCATTTGAAACTACTATTCTAGAGTG  160
NZ_LT635473_H_pylori_HE136/09       GCGTAGGCGGGATAGTCAGTCAGGTGTGAAATCCTATGGCTTAACCATAGAACTGCATTTGAAACTACTATTCTAGAGTG  160
NZ_LT635478_H_pylori_HE142/09       GCGTAGGCGGGATAGTCAGTCAGGTGTGAAATCCTATGGCTTAACCATAGAACTGCATTTGAAACTACTATTCTAGAGTG  160
NZ_LT635458_H_pylori_HE143/09       GCGTAGGCGGGATAGTCAGTCAGGTGTGAAATCCTATGGCTTAACCATAGAACTGCATTTGAAACTACTATTCTAGAGTG  160
NZ_LT635477_H_pylori_HE147/09       GCGTAGGCGGGATAGTCAGTCAGGTGTGAAATCCTATGGCTTAACCATAGAACTGCATTTGAAACTACTATTCTAGAGTG  160
NZ_LT635472_H_pylori_HE170/09       GCGTAGGCGGGATAGTCAGTCAGGTGTGAAATCCTATGGCTTAACCATAGAACTGCATTTGAAACTACTATTCTAGAGTG  160
NZ_LT635460_H_pylori_HE178/09       GCGTAGGCGGGATAGTCAGTCAGGTGTGAAATCCTATGGCTTAACCATAGAACTGCATTTGAAACTACTATTCTAGAGTG  160
NZ_LT838273_H_pylori_HE93/10_v1     GCGTAGGCGGGATAGTCAGTCAGGTGTGAAATCCTATGGCTTAACCATAGAACTGCATTTGAAACTACTATTCTAGAGTG  160
NZ_AP014710_H_pylori_DNA            GCGTAGGCGGGATAGTCAGTCAGGTGTGAAATCCTATGGCTTAACCATAGAACTGCATTTGAAACTACTATTCTAGAGTG  160
NZ_AP014710_H_pylori_DNA_copy2      GCGTAGGCGGGATAGTCAGTCAGGTGTGAAATCCTATGGCTTAACCATAGAACTGCATTTGAAACTACTATTCTAGAGTG  160
NZ_AP014712_H_pylori_DNA            GCGTAGGCGGGATAGTCAGTCAGGTGTGAAATCCTATGGCTTAACCATAGAACTGCATTTGAAACTACTATTCTAGAGTG  160
NZ_AP014712_H_pylori_DNA_copy2      GCGTAGGCGGGATAGTCAGTCAGGTGTGAAATCCTATGGCTTAACCATAGAACTGCATTTGAAACTACTATTCTAGAGTG  160
NC_017742_H_pylori_PeCan18_         GCGTAGGCGGGATAGTCAGTCAGGTGTGAAATCCTATGGCTTAACCATAGAACTGCATTTGAAACTACTATTCTAGAGTG  160
NC_021215.3_H_pylori_UM032          GCGTAGGCGGGATAGTCAGTCAGGTGTGAAATCCTATGGCTTAACCATAGAACTGCATTTGAAACTACTATTCTAGAGTG  160
NC_021882.2_H_pylori_UM298          GCGTAGGCGGGATAGTCAGTCAGGTGTGAAATCCTATGGCTTAACCATAGAACTGCATTTGAAACTACTATTCTAGAGTG  160
NC_021882.2_H_pylori_UM298_copy2    GCGTAGGCGGGATAGTCAGTCAGGTGTGAAATCCTATGGCTTAACCATAGAACTGCATTTGAAACTACTATTCTAGAGTG  160
NC_021216.3_H_pylori_UM299_copy2    GCGTAGGCGGGATAGTCAGTCAGGTGTGAAATCCTATGGCTTAACCATAGAACTGCATTTGAAACTACTATTCTAGAGTG  160
NC_017355_H_pylori_v225d_           GCGTAGGCGGGATAGTCAGTCAGGTGTGAAATCCTATGGCTTAACCATAGAACTGCATTTGAAACTACTATTCTAGAGTG  160
NC_017365_H_pylori_F30_DNA_         GCGTAGGCGGGATAGTCAGTCAGGTGTGAAATCCTATGGCTTAACCATAGAACTGCATTTGAAACTACTATTCTAGAGTG  160
NZ_CP024016_H_pylori_7.13_D1b       GCGTAGGCGGGATAGTCAGTCAGGTGTGAAATCCTATGGCTTAACCATAGAACTGCATTTGAAACTACTATTCTAGAGTG  160
CP024015_H_pylori_7.13_D1a_copy2    GCGTAGGCGGGATAGTCAGTCAGGTGTGAAATCCTATGGCTTAACCATAGAACTGCATTTGAAACTACTATTCTAGAGTG  160
CP024015_H_pylori_7.13_D1a          GCGTAGGCGGGATAGTCAGTCAGGTGTGAAATCCTATGGCTTAACCATAGAACTGCATTTGAAACTACTATTCTAGAGTG  160
NR_024570_E_coli_U_5/41             ACGCAGGCGGTTTGTTAAGTCAGATGTGAAATCCCCGGGCTCAACCTGGGAACTGCATCTGATACTGGCAAGCTTGAGTC  160
                                    0.......580.......590.......600.......610.......620.......630.......640.......65
```

CLUSTAL 2.0.11 MULTIPLE SEQUENCE ALIGNMENT


```
                                       * * *****  *** ******  ******* *** **** **********  *********   * ********* **
NZ_CP024016_H_pylori_7.13_D1b_copy2    TGGGAGAGGTAGGTGGAATTCTTGGTGTAGGGGTAAAATCCGTAGAGATCAAGAGGAATACTCATTGCGAAGGCGACCTG   240
NZ_CP028325_H_pylori_FDAARGOS_298      TGGGAGAGGTAGGTGGAATTCTTGGTGTAGGGGTAAAATCCGTAGAGATCAAGAGGAATACTCATTGCGAAGGCGACCTG   240
NZ_CP022409_H_pylori_G272              TGGGAGAGGTAGGTGGAATTCTTGGTGTAGGGGTAAAATCCGTAGAGATCAAGAGGAATACTCATTGCGAAGGCGACCTG   240
NZ_LT635459_H_pylori_HE132/09S         TGGGAGAGGTAGGTGGAATTCTTGGTGTAGGGGTAAAATCCGTAGAGATCAAGAGGAATACTCATTGCGAAGGCGACCTG   240
NZ_LT635471_H_pylori_HE141/09          TGGGAGAGGTAGGTGGAATTCTTGGTGTAGGGGTAAAATCCGTAGAGATCAAGAGGAATACTCATTGCGAAGGCGACCTG   240
NZ_LT635474_H_pylori_HE171/09          TGGGAGAGGTAGGTGGAATTCTTGGTGTAGGGGTAAAATCCGTAGAGATCAAGAGGAATACTCATTGCGAAGGCGACCTG   240
NC_017372_H_pylori_India7_             TGGGAGAGGTAGGTGGAATTCTTGGTGTAGGGGTAAAATCCGTAGAGATCAAGAGGAATACTCATTGCGAAGGCGACCTG   240
NZ_CP006822_H_pylori_oki128_           TGGGAGAGGTAGGTGGAATTCTTGGTGTAGGGGTAAAATCCGTAGAGATCAAGAGGAATACTCATTGCGAAGGCGACCTG   240
NC_021215.3_H_pylori_UM032_copy2       TGGGAGAGGTAGGTGGAATTCTTGGTGTAGGGGTAAAATCCGTAGAGATCAAGAGGAATACTCATTGCGAAGGCGACCTG   240
NC_021216.3_H_pylori_UM299             TGGGAGAGGTAGGTGGAATTCTTGGTGTAGGGGTAAAATCCGTAGAGATCAAGAGGAATACTCATTGCGAAGGCGACCTG   240
CP024017_H_pylori_7.13_D1c             TGGGAGAGGTAGGTGGAATTCTTGGTGTAGGGGTAAAATCCGTAGAGATCAAGAGGAATACTCATTGCGAAGGCGACCTG   240
CP024017_H_pylori_7.13_D1c_copy2       TGGGAGAGGTAGGTGGAATTCTTGGTGTAGGGGTAAAATCCGTAGAGATCAAGAGGAATACTCATTGCGAAGGCGACCTG   240
NZ_CP024021_H_pylori_7.13_D3a          TGGGAGAGGTAGGTGGAATTCTTGGTGTAGGGGTAAAATCCGTAGAGATCAAGAGGAATACTCATTGCGAAGGCGACCTG   240
NZ_CP024021_H_pylori_7.13_D3a_copy2    TGGGAGAGGTAGGTGGAATTCTTGGTGTAGGGGTAAAATCCGTAGAGATCAAGAGGAATACTCATTGCGAAGGCGACCTG   240
NZ_CP024022_H_pylori_7.13_D3b          TGGGAGAGGTAGGTGGAATTCTTGGTGTAGGGGTAAAATCCGTAGAGATCAAGAGGAATACTCATTGCGAAGGCGACCTG   240
NZ_CP024022_H_pylori_7.13_D3b_copy2    TGGGAGAGGTAGGTGGAATTCTTGGTGTAGGGGTAAAATCCGTAGAGATCAAGAGGAATACTCATTGCGAAGGCGACCTG   240
NZ_CP024023_H_pylori_7.13_D3c          TGGGAGAGGTAGGTGGAATTCTTGGTGTAGGGGTAAAATCCGTAGAGATCAAGAGGAATACTCATTGCGAAGGCGACCTG   240
NZ_CP024023_H_pylori_7.13_D3c_copy2    TGGGAGAGGTAGGTGGAATTCTTGGTGTAGGGGTAAAATCCGTAGAGATCAAGAGGAATACTCATTGCGAAGGCGACCTG   240
NZ_CP024071_H_pylori_7.13_R1a          TGGGAGAGGTAGGTGGAATTCTTGGTGTAGGGGTAAAATCCGTAGAGATCAAGAGGAATACTCATTGCGAAGGCGACCTG   240
NZ_CP024071_H_pylori_7.13_R1a_copy2    TGGGAGAGGTAGGTGGAATTCTTGGTGTAGGGGTAAAATCCGTAGAGATCAAGAGGAATACTCATTGCGAAGGCGACCTG   240
NZ_CP024072_H_pylori_7.13_R1b          TGGGAGAGGTAGGTGGAATTCTTGGTGTAGGGGTAAAATCCGTAGAGATCAAGAGGAATACTCATTGCGAAGGCGACCTG   240
NZ_CP024072_H_pylori_7.13_R1b_copy2    TGGGAGAGGTAGGTGGAATTCTTGGTGTAGGGGTAAAATCCGTAGAGATCAAGAGGAATACTCATTGCGAAGGCGACCTG   240
NZ_CP024073_H_pylori_7.13_R1c          TGGGAGAGGTAGGTGGAATTCTTGGTGTAGGGGTAAAATCCGTAGAGATCAAGAGGAATACTCATTGCGAAGGCGACCTG   240
NZ_CP024073_H_pylori_7.13_R1c_copy2    TGGGAGAGGTAGGTGGAATTCTTGGTGTAGGGGTAAAATCCGTAGAGATCAAGAGGAATACTCATTGCGAAGGCGACCTG   240
NZ_CP024074_H_pylori_7.13_R2a          TGGGAGAGGTAGGTGGAATTCTTGGTGTAGGGGTAAAATCCGTAGAGATCAAGAGGAATACTCATTGCGAAGGCGACCTG   240
NZ_CP024074_H_pylori_7.13_R2a_copy2    TGGGAGAGGTAGGTGGAATTCTTGGTGTAGGGGTAAAATCCGTAGAGATCAAGAGGAATACTCATTGCGAAGGCGACCTG   240
NZ_CP024075_H_pylori_7.13_R2b          TGGGAGAGGTAGGTGGAATTCTTGGTGTAGGGGTAAAATCCGTAGAGATCAAGAGGAATACTCATTGCGAAGGCGACCTG   240
NZ_CP024075_H_pylori_7.13_R2b_copy2    TGGGAGAGGTAGGTGGAATTCTTGGTGTAGGGGTAAAATCCGTAGAGATCAAGAGGAATACTCATTGCGAAGGCGACCTG   240
NZ_CP024076_H_pylori_7.13_R2c          TGGGAGAGGTAGGTGGAATTCTTGGTGTAGGGGTAAAATCCGTAGAGATCAAGAGGAATACTCATTGCGAAGGCGACCTG   240
NZ_CP024076_H_pylori_7.13_R2c_copy2    TGGGAGAGGTAGGTGGAATTCTTGGTGTAGGGGTAAAATCCGTAGAGATCAAGAGGAATACTCATTGCGAAGGCGACCTG   240
NZ_CP024077_H_pylori_7.13_R3a          TGGGAGAGGTAGGTGGAATTCTTGGTGTAGGGGTAAAATCCGTAGAGATCAAGAGGAATACTCATTGCGAAGGCGACCTG   240
NZ_CP024077_H_pylori_7.13_R3a_copy2    TGGGAGAGGTAGGTGGAATTCTTGGTGTAGGGGTAAAATCCGTAGAGATCAAGAGGAATACTCATTGCGAAGGCGACCTG   240
NZ_CP024078_H_pylori_7.13_R3b          TGGGAGAGGTAGGTGGAATTCTTGGTGTAGGGGTAAAATCCGTAGAGATCAAGAGGAATACTCATTGCGAAGGCGACCTG   240
NZ_CP024078_H_pylori_7.13_R3b_copy2    TGGGAGAGGTAGGTGGAATTCTTGGTGTAGGGGTAAAATCCGTAGAGATCAAGAGGAATACTCATTGCGAAGGCGACCTG   240
NZ_CP024079_H_pylori_7.13_R3c          TGGGAGAGGTAGGTGGAATTCTTGGTGTAGGGGTAAAATCCGTAGAGATCAAGAGGAATACTCATTGCGAAGGCGACCTG   240
NZ_CP024079_H_pylori_7.13_R3c_copy2    TGGGAGAGGTAGGTGGAATTCTTGGTGTAGGGGTAAAATCCGTAGAGATCAAGAGGAATACTCATTGCGAAGGCGACCTG   240
NC_019563_H_pylori_Aklavik86_          TGGGAGAGGTAGGTGGAATTCTTGGTGTAGGGGTAAAATCCGTAGAGATCAAGAGGAATACTCATTGCGAAGGCGACCTG   240
NZ_CP011485_H_pylori_ausabrJ05_        TGGGAGAGGTAGGTGGAATTCTTGGTGTAGGGGTAAAATCCGTAGAGATCAAGAGGAATACTCATTGCGAAGGCGACCTG   240
NC_014256_H_pylori_B8                  TGGGAGAGGTAGGTGGAATTCTTGGTGTAGGGGTAAAATCCGTAGAGATCAAGAGGAATACTCATTGCGAAGGCGACCTG   240
NC_014256_H_pylori_B8_copy2            TGGGAGAGGTAGGTGGAATTCTTGGTGTAGGGGTAAAATCCGTAGAGATCAAGAGGAATACTCATTGCGAAGGCGACCTG   240
NZ_LT837687_H_pylori_BCM-300           TGGGAGAGGTAGGTGGAATTCTTGGTGTAGGGGTAAAATCCGTAGAGATCAAGAGGAATACTCATTGCGAAGGCGACCTG   240
NC_022886_H_pylori_BM012A_             TGGGAGAGGTAGGTGGAATTCTTGGTGTAGGGGTAAAATCCGTAGAGATCAAGAGGAATACTCATTGCGAAGGCGACCTG   240
NZ_CP007605_H_pylori_BM012B_           TGGGAGAGGTAGGTGGAATTCTTGGTGTAGGGGTAAAATCCGTAGAGATCAAGAGGAATACTCATTGCGAAGGCGACCTG   240
NC_022911_H_pylori_BM012S_             TGGGAGAGGTAGGTGGAATTCTTGGTGTAGGGGTAAAATCCGTAGAGATCAAGAGGAATACTCATTGCGAAGGCGACCTG   240
NZ_CP011483_H_pylori_DU15_             TGGGAGAGGTAGGTGGAATTCTTGGTGTAGGGGTAAAATCCGTAGAGATCAAGAGGAATACTCATTGCGAAGGCGACCTG   240
NZ_CP022409_H_pylori_G272_copy2        TGGGAGAGGTAGGTGGAATTCTTGGTGTAGGGGTAAAATCCGTAGAGATCAAGAGGAATACTCATTGCGAAGGCGACCTG   240
NZ_LT635456_H_pylori_HE101/09          TGGGAGAGGTAGGTGGAATTCTTGGTGTAGGGGTAAAATCCGTAGAGATCAAGAGGAATACTCATTGCGAAGGCGACCTG   240
NZ_LT635476_H_pylori_HE134/09          TGGGAGAGGTAGGTGGAATTCTTGGTGTAGGGGTAAAATCCGTAGAGATCAAGAGGAATACTCATTGCGAAGGCGACCTG   240
NZ_LT635473_H_pylori_HE136/09          TGGGAGAGGTAGGTGGAATTCTTGGTGTAGGGGTAAAATCCGTAGAGATCAAGAGGAATACTCATTGCGAAGGCGACCTG   240
NZ_LT635478_H_pylori_HE142/09          TGGGAGAGGTAGGTGGAATTCTTGGTGTAGGGGTAAAATCCGTAGAGATCAAGAGGAATACTCATTGCGAAGGCGACCTG   240
NZ_LT635458_H_pylori_HE143/09          TGGGAGAGGTAGGTGGAATTCTTGGTGTAGGGGTAAAATCCGTAGAGATCAAGAGGAATACTCATTGCGAAGGCGACCTG   240
NZ_LT635477_H_pylori_HE147/09          TGGGAGAGGTAGGTGGAATTCTTGGTGTAGGGGTAAAATCCGTAGAGATCAAGAGGAATACTCATTGCGAAGGCGACCTG   240
NZ_LT635472_H_pylori_HE170/09          TGGGAGAGGTAGGTGGAATTCTTGGTGTAGGGGTAAAATCCGTAGAGATCAAGAGGAATACTCATTGCGAAGGCGACCTG   240
NZ_LT635460_H_pylori_HE178/09          TGGGAGAGGTAGGTGGAATTCTTGGTGTAGGGGTAAAATCCGTAGAGATCAAGAGGAATACTCATTGCGAAGGCGACCTG   240
NZ_LT838273_H_pylori_HE93/10_v1        TGGGAGAGGTAGGTGGAATTCTTGGTGTAGGGGTAAAATCCGTAGAGATCAAGAGGAATACTCATTGCGAAGGCGACCTG   240
NZ_AP014710_H_pylori_DNA               TGGGAGAGGTAGGTGGAATTCTTGGTGTAGGGGTAAAATCCGTAGAGATCAAGAGGAATACTCATTGCGAAGGCGACCTG   240
NZ_AP014710_H_pylori_DNA_copy2         TGGGAGAGGTAGGTGGAATTCTTGGTGTAGGGGTAAAATCCGTAGAGATCAAGAGGAATACTCATTGCGAAGGCGACCTG   240
NZ_AP014712_H_pylori_DNA               TGGGAGAGGTAGGTGGAATTCTTGGTGTAGGGGTAAAATCCGTAGAGATCAAGAGGAATACTCATTGCGAAGGCGACCTG   240
NZ_AP014712_H_pylori_DNA_copy2         TGGGAGAGGTAGGTGGAATTCTTGGTGTAGGGGTAAAATCCGTAGAGATCAAGAGGAATACTCATTGCGAAGGCGACCTG   240
NC_017742_H_pylori_PeCan18_            TGGGAGAGGTAGGTGGAATTCTTGGTGTAGGGGTAAAATCCGTAGAGATCAAGAGGAATACTCATTGCGAAGGCGACCTG   240
NC_021215.3_H_pylori_UM032             TGGGAGAGGTAGGTGGAATTCTTGGTGTAGGGGTAAAATCCGTAGAGATCAAGAGGAATACTCATTGCGAAGGCGACCTG   240
NC_021882.2_H_pylori_UM298             TGGGAGAGGTAGGTGGAATTCTTGGTGTAGGGGTAAAATCCGTAGAGATCAAGAGGAATACTCATTGCGAAGGCGACCTG   240
NC_021882.2_H_pylori_UM298_copy2       TGGGAGAGGTAGGTGGAATTCTTGGTGTAGGGGTAAAATCCGTAGAGATCAAGAGGAATACTCATTGCGAAGGCGACCTG   240
NC_021216.3_H_pylori_UM299_copy2       TGGGAGAGGTAGGTGGAATTCTTGGTGTAGGGGTAAAATCCGTAGAGATCAAGAGGAATACTCATTGCGAAGGCGACCTG   240
NC_017355_H_pylori_v225d_              TGGGAGAGGTAGGTGGAATTCTTGGTGTAGGGGTAAAATCCGTAGAGATCAAGAGGAATACTCATTGCGAAGGCGACCTG   240
NC_017365_H_pylori_F30_DNA_            TGGGAGAGGTAGGTGGAATTCTTGGTGTAGGGGTAAAATCCGTAGAGATCAAGAGGAATACTCATTGCGAAGGCGACCTG   240
NZ_CP024016_H_pylori_7.13_D1b          TGGGAGAGGTAGGTGGAATTCTTGGTGTAGGGGTAAAATCCGTAGAGATCAAGAGGAATACTCATTGCGAAGGCGACCTG   240
CP024015_H_pylori_7.13_D1a_copy2       TGGGAGAGGTAGGTGGAATTCTTGGTGTAGGGGTAAAATCCGTAGAGATCAAGAGGAATACTCATTGCGAAGGCGACCTG   240
CP024015_H_pylori_7.13_D1a             TGGGAGAGGTAGGTGGAATTCTTGGTGTAGGGGTAAAATCCGTAGAGATCAAGAGGAATACTCATTGCGAAGGCGACCTG   240
NR_024570_E_coli_U_5/41                TCGTAGAGGGGGGTAGAATTCCAGGTGTAGCGGTGAAATGCGTAGAGATCTGGAGGAATACCGGTGGCGAAGGCGGCCCC   240
                                       0.......660.......670.......680.......690.......700.......710.......720.......73
```

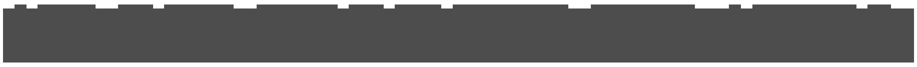

CLUSTAL 2.0.11 MULTIPLE SEQUENCE ALIGNMENT


```
                                    *****  *  ********* *  *  ********************************************
NZ_CP024016_H_pylori_7.13_D1b_copy2 CTGGAACATTACTGACGCTGATTGCGCGAAAGCGTGGGGAGCAAACAGGATTAGATACCCTGGTAGTCC-----------   320
NZ_CP028325_H_pylori_FDAARGOS_298   CTGGAACATTACTGACGCTGATTGCGCGAAAGCGTGGGGAGCAAACAGGATTAGATACCCTGGTAGTCC-----------   320
NZ_CP022409_H_pylori_G272           CTGGAACATTACTGACGCTGATTGCGCGAAAGCGTGGGGAGCAAACAGGATTAGATACCCTGGTAGTCC-----------   320
NZ_LT635459_H_pylori_HE132/09S      CTGGAACATTACTGACGCTGATTGCGCGAAAGCGTGGGGAGCAAACAGGATTAGATACCCTGGTAGTCC-----------   320
NZ_LT635471_H_pylori_HE141/09       CTGGAACATTACTGACGCTGATTGCGCGAAAGCGTGGGGAGCAAACAGGATTAGATACCCTGGTAGTCC-----------   320
NZ_LT635474_H_pylori_HE171/09       CTGGAACATTACTGACGCTGATTGCGCGAAAGCGTGGGGAGCAAACAGGATTAGATACCCTGGTAGTCC-----------   320
NC_017372_H_pylori_India7_          CTGGAACATTACTGACGCTGATTGCGCGAAAGCGTGGGGAGCAAACAGGATTAGATACCCTGGTAGTCC-----------   320
NZ_CP006822_H_pylori_oki128_        CTGGAACATTACTGACGCTGATTGCGCGAAAGCGTGGGGAGCAAACAGGATTAGATACCCTGGTAGTCC-----------   320
NC_021215.3_H_pylori_UM032_copy2    CTGGAACATTACTGACGCTGATTGCGCGAAAGCGTGGGGAGCAAACAGGATTAGATACCCTGGTAGTCC-----------   320
NC_021216.3_H_pylori_UM299          CTGGAACATTACTGACGCTGATTGCGCGAAAGCGTGGGGAGCAAACAGGATTAGATACCCTGGTAGTCC-----------   320
CP024017_H_pylori_7.13_D1c          CTGGAACATTACTGACGCTGATTGCGCGAAAGCGTGGGGAGCAAACAGGATTAGATACCCTGGTAGTCC-----------   320
CP024017_H_pylori_7.13_D1c_copy2    CTGGAACATTACTGACGCTGATTGCGCGAAAGCGTGGGGAGCAAACAGGATTAGATACCCTGGTAGTCC-----------   320
NZ_CP024021_H_pylori_7.13_D3a       CTGGAACATTACTGACGCTGATTGCGCGAAAGCGTGGGGAGCAAACAGGATTAGATACCCTGGTAGTCC-----------   320
NZ_CP024021_H_pylori_7.13_D3a_copy2 CTGGAACATTACTGACGCTGATTGCGCGAAAGCGTGGGGAGCAAACAGGATTAGATACCCTGGTAGTCC-----------   320
NZ_CP024022_H_pylori_7.13_D3b       CTGGAACATTACTGACGCTGATTGCGCGAAAGCGTGGGGAGCAAACAGGATTAGATACCCTGGTAGTCC-----------   320
NZ_CP024022_H_pylori_7.13_D3b_copy2 CTGGAACATTACTGACGCTGATTGCGCGAAAGCGTGGGGAGCAAACAGGATTAGATACCCTGGTAGTCC-----------   320
NZ_CP024023_H_pylori_7.13_D3c       CTGGAACATTACTGACGCTGATTGCGCGAAAGCGTGGGGAGCAAACAGGATTAGATACCCTGGTAGTCC-----------   320
NZ_CP024023_H_pylori_7.13_D3c_copy2 CTGGAACATTACTGACGCTGATTGCGCGAAAGCGTGGGGAGCAAACAGGATTAGATACCCTGGTAGTCC-----------   320
NZ_CP024071_H_pylori_7.13_R1a       CTGGAACATTACTGACGCTGATTGCGCGAAAGCGTGGGGAGCAAACAGGATTAGATACCCTGGTAGTCC-----------   320
NZ_CP024071_H_pylori_7.13_R1a_copy2 CTGGAACATTACTGACGCTGATTGCGCGAAAGCGTGGGGAGCAAACAGGATTAGATACCCTGGTAGTCC-----------   320
NZ_CP024072_H_pylori_7.13_R1b       CTGGAACATTACTGACGCTGATTGCGCGAAAGCGTGGGGAGCAAACAGGATTAGATACCCTGGTAGTCC-----------   320
NZ_CP024072_H_pylori_7.13_R1b_copy2 CTGGAACATTACTGACGCTGATTGCGCGAAAGCGTGGGGAGCAAACAGGATTAGATACCCTGGTAGTCC-----------   320
NZ_CP024073_H_pylori_7.13_R1c       CTGGAACATTACTGACGCTGATTGCGCGAAAGCGTGGGGAGCAAACAGGATTAGATACCCTGGTAGTCC-----------   320
NZ_CP024073_H_pylori_7.13_R1c_copy2 CTGGAACATTACTGACGCTGATTGCGCGAAAGCGTGGGGAGCAAACAGGATTAGATACCCTGGTAGTCC-----------   320
NZ_CP024074_H_pylori_7.13_R2a       CTGGAACATTACTGACGCTGATTGCGCGAAAGCGTGGGGAGCAAACAGGATTAGATACCCTGGTAGTCC-----------   320
NZ_CP024074_H_pylori_7.13_R2a_copy2 CTGGAACATTACTGACGCTGATTGCGCGAAAGCGTGGGGAGCAAACAGGATTAGATACCCTGGTAGTCC-----------   320
NZ_CP024075_H_pylori_7.13_R2b       CTGGAACATTACTGACGCTGATTGCGCGAAAGCGTGGGGAGCAAACAGGATTAGATACCCTGGTAGTCC-----------   320
NZ_CP024075_H_pylori_7.13_R2b_copy2 CTGGAACATTACTGACGCTGATTGCGCGAAAGCGTGGGGAGCAAACAGGATTAGATACCCTGGTAGTCC-----------   320
NZ_CP024076_H_pylori_7.13_R2c       CTGGAACATTACTGACGCTGATTGCGCGAAAGCGTGGGGAGCAAACAGGATTAGATACCCTGGTAGTCC-----------   320
NZ_CP024076_H_pylori_7.13_R2c_copy2 CTGGAACATTACTGACGCTGATTGCGCGAAAGCGTGGGGAGCAAACAGGATTAGATACCCTGGTAGTCC-----------   320
NZ_CP024077_H_pylori_7.13_R3a       CTGGAACATTACTGACGCTGATTGCGCGAAAGCGTGGGGAGCAAACAGGATTAGATACCCTGGTAGTCC-----------   320
NZ_CP024077_H_pylori_7.13_R3a_copy2 CTGGAACATTACTGACGCTGATTGCGCGAAAGCGTGGGGAGCAAACAGGATTAGATACCCTGGTAGTCC-----------   320
NZ_CP024078_H_pylori_7.13_R3b       CTGGAACATTACTGACGCTGATTGCGCGAAAGCGTGGGGAGCAAACAGGATTAGATACCCTGGTAGTCC-----------   320
NZ_CP024078_H_pylori_7.13_R3b_copy2 CTGGAACATTACTGACGCTGATTGCGCGAAAGCGTGGGGAGCAAACAGGATTAGATACCCTGGTAGTCC-----------   320
NZ_CP024079_H_pylori_7.13_R3c       CTGGAACATTACTGACGCTGATTGCGCGAAAGCGTGGGGAGCAAACAGGATTAGATACCCTGGTAGTCC-----------   320
NZ_CP024079_H_pylori_7.13_R3c_copy2 CTGGAACATTACTGACGCTGATTGCGCGAAAGCGTGGGGAGCAAACAGGATTAGATACCCTGGTAGTCC-----------   320
NC_019563_H_pylori_Aklavik86_       CTGGAACATTACTGACGCTGATTGCGCGAAAGCGTGGGGAGCAAACAGGATTAGATACCCTGGTAGTCC-----------   320
NZ_CP011485_H_pylori_ausabrJ05_     CTGGAACATTACTGACGCTGATTGCGCGAAAGCGTGGGGAGCAAACAGGATTAGATACCCTGGTAGTCC-----------   320
NC_014256_H_pylori_B8               CTGGAACATTACTGACGCTGATTGCGCGAAAGCGTGGGGAGCAAACAGGATTAGATACCCTGGTAGTCC-----------   320
NC_014256_H_pylori_B8_copy2         CTGGAACATTACTGACGCTGATTGCGCGAAAGCGTGGGGAGCAAACAGGATTAGATACCCTGGTAGTCC-----------   320
NZ_LT837687_H_pylori_BCM-300        CTGGAACATTACTGACGCTGATTGCGCGAAAGCGTGGGGAGCAAACAGGATTAGATACCCTGGTAGTCC-----------   320
NC_022886_H_pylori_BM012A_          CTGGAACATTACTGACGCTGATTGCGCGAAAGCGTGGGGAGCAAACAGGATTAGATACCCTGGTAGTCC-----------   320
NZ_CP007605_H_pylori_BM012B_        CTGGAACATTACTGACGCTGATTGCGCGAAAGCGTGGGGAGCAAACAGGATTAGATACCCTGGTAGTCC-----------   320
NC_022911_H_pylori_BM012S_          CTGGAACATTACTGACGCTGATTGCGCGAAAGCGTGGGGAGCAAACAGGATTAGATACCCTGGTAGTCC-----------   320
NZ_CP011483_H_pylori_DU15_          CTGGAACATTACTGACGCTGATTGCGCGAAAGCGTGGGGAGCAAACAGGATTAGATACCCTGGTAGTCC-----------   320
NZ_CP022409_H_pylori_G272_copy2     CTGGAACATTACTGACGCTGATTGCGCGAAAGCGTGGGGAGCAAACAGGATTAGATACCCTGGTAGTCC-----------   320
NZ_LT635456_H_pylori_HE101/09       CTGGAACATTACTGACGCTGATTGCGCGAAAGCGTGGGGAGCAAACAGGATTAGATACCCTGGTAGTCC-----------   320
NZ_LT635476_H_pylori_HE134/09       CTGGAACATTACTGACGCTGATTGCGCGAAAGCGTGGGGAGCAAACAGGATTAGATACCCTGGTAGTCC-----------   320
NZ_LT635473_H_pylori_HE136/09       CTGGAACATTACTGACGCTGATTGCGCGAAAGCGTGGGGAGCAAACAGGATTAGATACCCTGGTAGTCC-----------   320
NZ_LT635478_H_pylori_HE142/09       CTGGAACATTACTGACGCTGATTGCGCGAAAGCGTGGGGAGCAAACAGGATTAGATACCCTGGTAGTCC-----------   320
NZ_LT635458_H_pylori_HE143/09       CTGGAACATTACTGACGCTGATTGCGCGAAAGCGTGGGGAGCAAACAGGATTAGATACCCTGGTAGTCC-----------   320
NZ_LT635477_H_pylori_HE147/09       CTGGAACATTACTGACGCTGATTGCGCGAAAGCGTGGGGAGCAAACAGGATTAGATACCCTGGTAGTCC-----------   320
NZ_LT635472_H_pylori_HE170/09       CTGGAACATTACTGACGCTGATTGCGCGAAAGCGTGGGGAGCAAACAGGATTAGATACCCTGGTAGTCC-----------   320
NZ_LT635460_H_pylori_HE178/09       CTGGAACATTACTGACGCTGATTGCGCGAAAGCGTGGGGAGCAAACAGGATTAGATACCCTGGTAGTCC-----------   320
NZ_LT838273_H_pylori_HE93/10_v1     CTGGAACATTACTGACGCTGATTGCGCGAAAGCGTGGGGAGCAAACAGGATTAGATACCCTGGTAGTCC-----------   320
NZ_AP014710_H_pylori_DNA            CTGGAACATTACTGACGCTGATTGCGCGAAAGCGTGGGGAGCAAACAGGATTAGATACCCTGGTAGTCC-----------   320
NZ_AP014710_H_pylori_DNA_copy2      CTGGAACATTACTGACGCTGATTGCGCGAAAGCGTGGGGAGCAAACAGGATTAGATACCCTGGTAGTCC-----------   320
NZ_AP014712_H_pylori_DNA            CTGGAACATTACTGACGCTGATTGCGCGAAAGCGTGGGGAGCAAACAGGATTAGATACCCTGGTAGTCC-----------   320
NZ_AP014712_H_pylori_DNA_copy2      CTGGAACATTACTGACGCTGATTGCGCGAAAGCGTGGGGAGCAAACAGGATTAGATACCCTGGTAGTCC-----------   320
NC_017742_H_pylori_PeCan18_         CTGGAACATTACTGACGCTGATTGCGCGAAAGCGTGGGGAGCAAACAGGATTAGATACCCTGGTAGTCC-----------   320
NC_021215.3_H_pylori_UM032          CTGGAACATTACTGACGCTGATTGCGCGAAAGCGTGGGGAGCAAACAGGATTAGATACCCTGGTAGTCC-----------   320
NC_021882.2_H_pylori_UM298          CTGGAACATTACTGACGCTGATTGCGCGAAAGCGTGGGGAGCAAACAGGATTAGATACCCTGGTAGTCC-----------   320
NC_021882.2_H_pylori_UM298_copy2    CTGGAACATTACTGACGCTGATTGCGCGAAAGCGTGGGGAGCAAACAGGATTAGATACCCTGGTAGTCC-----------   320
NC_021216.3_H_pylori_UM299_copy2    CTGGAACATTACTGACGCTGATTGCGCGAAAGCGTGGGGAGCAAACAGGATTAGATACCCTGGTAGTCC-----------   320
NC_017355_H_pylori_v225d_           CTGGAACATTACTGACGCTGATTGCGCGAAAGCGTGGGGAGCAAACAGGATTAGATACCCTGGTAGTCC-----------   320
NC_017365_H_pylori_F30_DNA_         CTGGAACATTACTGACGCTGATTGCACGAAAGCGTGGGGAGCAAACAGGATTAGATACCCTGGTAGTCC-----------   320
NZ_CP024016_H_pylori_7.13_D1b       CTGGAACATTACTGACGCTGATTGCGCGAAAGCGTGGGGAGCAAACAGGATTAGATACCCTGGTAGTCC-----------   320
CP024015_H_pylori_7.13_D1a_copy2    CTGGAACATTACTGACGCTGATTGCGCGAAAGCGTGGGGAGCAAACAGGATTAGATACCCTGGTAGTCC-----------   320
CP024015_H_pylori_7.13_D1a          CTGGAACATTACTGACGCTGATTGCGCGAAAGCGTGGGGAGCAAACAGGATTAGATACCCTGGTAGTCC-----------   320
NR_024570_E_coli_U_5/41             CTGGACGAAGACTGACGCTCAG-GTGCGAAAGCGTGGGGAGCAAACAGGATTAGATACCCTGGTAGTCCACGCCGTAAAC   320
                                    0.......740.......750.......760.......770.......780.......790.......800.......81
```

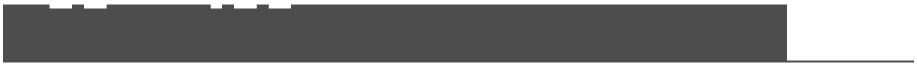

```
NZ_CP024016_H_pylori_7.13_D1b_copy2 -    321
NZ_CP028325_H_pylori_FDAARGOS_298   -    321
NZ_CP022409_H_pylori_G272           -    321
NZ_LT635459_H_pylori_HE132/09S      -    321
NZ_LT635471_H_pylori_HE141/09       -    321
NZ_LT635474_H_pylori_HE171/09       -    321
NC_017372_H_pylori_India7_          -    321
NZ_CP006822_H_pylori_oki128_        -    321
NC_021215.3_H_pylori_UM032_copy2    -    321
NC_021216.3_H_pylori_UM299          -    321
CP024017_H_pylori_7.13_D1c          -    321
CP024017_H_pylori_7.13_D1c_copy2    -    321
NZ_CP024021_H_pylori_7.13_D3a       -    321
NZ_CP024021_H_pylori_7.13_D3a_copy2 -    321
NZ_CP024022_H_pylori_7.13_D3b       -    321
NZ_CP024022_H_pylori_7.13_D3b_copy2 -    321
NZ_CP024023_H_pylori_7.13_D3c       -    321
NZ_CP024023_H_pylori_7.13_D3c_copy2 -    321
NZ_CP024071_H_pylori_7.13_R1a       -    321
NZ_CP024071_H_pylori_7.13_R1a_copy2 -    321
NZ_CP024072_H_pylori_7.13_R1b       -    321
NZ_CP024072_H_pylori_7.13_R1b_copy2 -    321
NZ_CP024073_H_pylori_7.13_R1c       -    321
NZ_CP024073_H_pylori_7.13_R1c_copy2 -    321
NZ_CP024074_H_pylori_7.13_R2a       -    321
NZ_CP024074_H_pylori_7.13_R2a_copy2 -    321
NZ_CP024075_H_pylori_7.13_R2b       -    321
NZ_CP024075_H_pylori_7.13_R2b_copy2 -    321
NZ_CP024076_H_pylori_7.13_R2c       -    321
NZ_CP024076_H_pylori_7.13_R2c_copy2 -    321
NZ_CP024077_H_pylori_7.13_R3a       -    321
NZ_CP024077_H_pylori_7.13_R3a_copy2 -    321
NZ_CP024078_H_pylori_7.13_R3b       -    321
NZ_CP024078_H_pylori_7.13_R3b_copy2 -    321
NZ_CP024079_H_pylori_7.13_R3c       -    321
NZ_CP024079_H_pylori_7.13_R3c_copy2 -    321
NC_019563_H_pylori_Aklavik86_       -    321
NZ_CP011485_H_pylori_ausabrJ05_     -    321
NC_014256_H_pylori_B8               -    321
NC_014256_H_pylori_B8_copy2         -    321
NZ_LT837687_H_pylori_BCM-300        -    321
NC_022886_H_pylori_BM012A_          -    321
NZ_CP007605_H_pylori_BM012B_        -    321
NC_022911_H_pylori_BM012S_          -    321
NZ_CP011483_H_pylori_DU15_          -    321
NZ_CP022409_H_pylori_G272_copy2     -    321
NZ_LT635456_H_pylori_HE101/09       -    321
NZ_LT635476_H_pylori_HE134/09       -    321
NZ_LT635473_H_pylori_HE136/09       -    321
NZ_LT635478_H_pylori_HE142/09       -    321
NZ_LT635458_H_pylori_HE143/09       -    321
NZ_LT635477_H_pylori_HE147/09       -    321
NZ_LT635472_H_pylori_HE170/09       -    321
NZ_LT635460_H_pylori_HE178/09       -    321
NZ_LT838273_H_pylori_HE93/10_v1     -    321
NZ_AP014710_H_pylori_DNA            -    321
NZ_AP014710_H_pylori_DNA_copy2      -    321
NZ_AP014712_H_pylori_DNA            -    321
NZ_AP014712_H_pylori_DNA_copy2      -    321
NC_017742_H_pylori_PeCan18_         -    321
NC_021215.3_H_pylori_UM032          -    321
NC_021882.2_H_pylori_UM298          -    321
NC_021882.2_H_pylori_UM298_copy2    -    321
NC_021216.3_H_pylori_UM299_copy2    -    321
NC_017355_H_pylori_v225d_           -    321
NC_017365_H_pylori_F30_DNA_         -    321
NZ_CP024016_H_pylori_7.13_D1b       -    321
CP024015_H_pylori_7.13_D1a_copy2    -    321
CP024015_H_pylori_7.13_D1a          -    321
NR_024570_E_coli_U_5/41             G    321
                                    0
```
